# Soluble PD-L1 reprograms blood monocytes to prevent cerebral edema and facilitate recovery after ischemic stroke

**DOI:** 10.1016/j.bbi.2023.12.007

**Published:** 2023-12-07

**Authors:** Jennifer E. Kim, Ryan P. Lee, Eli Yazigi, Lyla Atta, James Feghali, Ayush Pant, Aanchal Jain, Idan Levitan, Eileen Kim, Kisha Patel, Nivedha Kannapadi, Pavan Shah, Adnan Bibic, Zhipeng Hou, Justin M. Caplan, L. Fernando Gonzalez, Judy Huang, Risheng Xu, Jean Fan, Betty Tyler, Henry Brem, Vassiliki A. Boussiotis, Lauren Jantzie, Shenandoah Robinson, Raymond C. Koehler, Michael Lim, Rafael J. Tamargo, Christopher M. Jackson

**Affiliations:** a Department of Neurosurgery, Johns Hopkins University School of Medicine, Baltimore, MD, the United States of America; b Department of Biomedical Engineering, Johns Hopkins University, Baltimore, MD, the United States of America; c Center for Computational Biology, Whiting School of Engineering, Johns Hopkins University, Baltimore, MD, the United States of America; d Medical Scientist Training Program, Johns Hopkins University School of Medicine, Baltimore, MD, the United States of America; e Department of Neurosurgery, Rabin Medical Center, Sackler Medical School, Petah Tikva, Israel; f F.M. Kirby Research Center for Functional Brain Imaging, Kennedy Krieger Institute, Baltimore, MD, the United States of America; g The Russell H. Morgan Department of Radiology and Radiological Science, Johns Hopkins University School of Medicine, Baltimore, MD, the United States of America; h Department of Radiology, School of Medicine, Johns Hopkins University, Baltimore, MD, the United States of America; i Division of Hematology-Oncology, Beth Israel Deaconess Medical Center, Harvard Medical School, Boston, MA, the United States of America; j Departments of Pediatrics, Johns Hopkins University School of Medicine, Maryland, the United States of America; k Kennedy Krieger Institute, Maryland, the United States of America; l Department of Neurology, Johns Hopkins University School of Medicine, Maryland, the United States of America; m Departments of Anesthesiology and Critical Care Medicine, Johns Hopkins University, School of Medicine, Baltimore, MD, the United States of America; n Department of Neurosurgery, Stanford University School of Medicine, Stanford, CA, the United States of America

**Keywords:** Ischemic stroke, Immune checkpoints, sPD-L1, Monocytes, Inflammation, Large vessel occlusion, MCAO

## Abstract

Acute cerebral ischemia triggers a profound inflammatory response. While macrophages polarized to an M2-like phenotype clear debris and facilitate tissue repair, aberrant or prolonged macrophage activation is counterproductive to recovery. The inhibitory immune checkpoint Programmed Cell Death Protein 1 (PD-1) is upregulated on macrophage precursors (monocytes) in the blood after acute cerebrovascular injury. To investigate the therapeutic potential of PD-1 activation, we immunophenotyped circulating monocytes from patients and found that PD-1 expression was upregulated in the acute period after stroke. Murine studies using a temporary middle cerebral artery (MCA) occlusion (MCAO) model showed that intraperitoneal administration of soluble Programmed Death Ligand-1 (sPD-L1) significantly decreased brain edema and improved overall survival. Mice receiving sPD-L1 also had higher performance scores short-term, and more closely resembled sham animals on assessments of long-term functional recovery. These clinical and radiographic benefits were abrogated in global and myeloid-specific PD-1 knockout animals, confirming PD-1+ monocytes as the therapeutic target of sPD-L1. Single-cell RNA sequencing revealed that treatment skewed monocyte maturation to a non-classical Ly6C^lo^, CD43^hi^, PD-L1+ phenotype. These data support peripheral activation of PD-1 on inflammatory monocytes as a therapeutic strategy to treat neuroinflammation after acute ischemic stroke.

## 1. Introduction

Stroke is the second leading cause of death worldwide (Donkor, 2018) and a leading cause of long-term disability (Feigin et al., 2017). An estimated 87 % of strokes are ischemic (Virani et al., 2020), and large vessel occlusions (LVOs)—defined as occlusion of the internal carotid artery (ICA), proximal middle cerebral artery (M1, M2), proximal anterior cerebral artery (A1, A2), vertebral artery, basilar artery, or proximal posterior cerebral artery (P1, P2)—account for 20–40 % of acute ischemic strokes (Malhotra et al., 2017; Smith et al., 2009). Primary injury in acute ischemic stroke results from rapid cell death in the infarct core due to sudden disruption of cerebral perfusion. Secondary injury is initiated when dying neurons in the ischemic core undergo metabolic failure, resulting in cytotoxic edema (Liang et al., 2007; Stokum et al., 2016; Simard et al., 2007) and release damage-associated molecular patterns (DAMPs). DAMPs trigger activation, recruitment, and trafficking of immune cells into the ischemic core and penumbra, with maximum accumulation of neutrophils and monocytes during the first three to seven days post-ictus (Gelderblom et al., 2009; Qiu et al., 2021). This immune cell infiltration contributes to endothelial dysfunction of the cerebral microvasculature, causes blood brain barrier (BBB) permeabilization, and generates the driving force for vasogenic (*trans*-vascular) edema. Unlike cytotoxic edema, which is due to early intracellular fluid shifts, vasogenic edema is caused by extracellular water extravasation from the vascular compartment down ion gradients, and results in increased volume or swelling of the brain tissues (Stokum et al., 2016; Simard et al., 2007). The resulting mass effect within the fixed cranial vault causes elevated intracranial pressure and brain shift, with a high incidence of permanent injury or death (Hofmeijer et al., 2008).

LVOs are associated with larger infarct volumes and contribute to a disproportionately higher rate of post-stroke dependence (61.6 %) and mortality (95.6 %) (Malhotra et al., 2017). The primary intervention is expeditious restoration of cerebral blood flow using intravenous thrombolytics or mechanical thrombectomy to minimize ischemic cell death. The care that follows is supportive. For patients with lifethreatening cerebral edema, surgical decompression affords a survival benefit, but does not improve functional outcomes, and carries additional morbidity (Lin and Frontera, 2021). Attempts to inhibit cerebral inflammation after stroke, including use of steroids, have been unsuccessful due to lack of selectivity and off-target effects (Qizilbash et al., 2002). Targeted anti-inflammatory approaches are of clinical interest for their potential to prevent secondary inflammatory injury following LVO.

Immune checkpoints and their ligands are expressed on activated immune cells and protect against aberrant inflammation in healthy tissues or restrain overly robust responses that persist after a threat has been eliminated (Pardoll, 2012). PD-1 is upregulated on immune cells upon activation while its ligands are highly expressed in damaged tissues (Barber et al., 2006; Keir et al., 2008). PD-1 and PD-L1 blocking antibodies have been successfully used in advanced cancers to amplify antitumor immune responses (Yi et al., 2022). More recently, PD-1 agonism to treat chronic inflammation has gained traction as a phase 2a trial of a PD-1 agonist antibody for rheumatoid arthritis generated positive results (Gravallese and Thomas, 2023). It is not yet clear if PD-1 agonism can be used to treat acute inflammation, which is primarily driven by innate immune cells, however, a growing body of evidence suggests that the PD-1 pathway plays a role in ischemic CNS injury (Wicks et al., 2022; Jin et al., 2010); however, there are conflicting data regarding the outcomes of PD-1 activation. In a middle cerebral artery occlusion (MCAO) model, PD-1 knockout mice had larger infarcts and worse functional outcomes when compared with wild-type (Ren et al., 2011). Conversely, PD-L1 blockade decreased infarct volumes and improved outcomes after MCAO (Bodhankar et al., 2015). These conflicting results may, in part, be due to location of PD-1/PD-L1 interactions as systemic administration of sPD-L1 decreases inflammation after ICH (Han et al., 2017). We previously reported that PD-1 expression on circulating monocytes in patients with ruptured cerebral aneurysms correlated with cerebral vasospasm, while systemic sPD-L1 administration prevented vasospasm after subarachnoid hemorrhage in an ICA perforation model by inhibiting ingress of PD-1+, Ly6C^hi^, CCR2^hi^ inflammatory monocytes into the brain (Jackson et al., 2021). Here we explore the potential of using sPD-L1 to activate PD-1 on peripheral monocytes and limit secondary inflammatory injury after LVO.

## 2. Materials and methods

### 2.1. Study design

This study was designed to investigate the anti-inflammatory effects of sPD-L1 in the setting of acute ischemic stroke. Using a well-established murine model of LVO and MCA distribution infarct, we evaluated the role of PD-1 expressing monocytes in neuroinflammation and cerebral edema and as potential targets of therapeutic intervention. We used high-resolution MRI, wet versus dry weight measurements, flow cytometry, standardized behavioral tests, scRNA-seq, and a Seahorse bioanalyzer. Human blood samples were collected under the institutional review board (IRB) protocol IRB00258362. All animal experiments were performed in accordance with protocols approved by the Institutional Animal Care and Use Committee (IACUC).

### 2.2. Human patient sample collection

A total of 14 adult (>18 years) patients who presented to the Johns Hopkins Hospital between January 2021 and January 2023 with acute LVO of the middle cerebral artery were enrolled into the study. The study was approved by the IRB and informed consent was obtained from participating patients or their next of kin as appropriate. Recorded characteristics included patient sex, time of last known well, presentation time, the Alberta stroke program early CT score (ASPECTS) on arrival, LVO location including vessel involved and laterality, National Institutes of Health Stroke Scale (NIHSS) score on arrival, whether the patient underwent mechanical thrombectomy, timing of recanalization, Thrombolysis in Cerebral Infarction (TICI) score, and total infarct volume and edema volume. Volumetric measurements were calculated using manual segmentation. Patients with pre-existing active systemic infections such as COVID-19 or hepatitis, or who underwent emergent decompressive craniectomy or any surgical intervention other than mechanical thrombectomy during the period of blood sample collection were excluded. Patients who presented with small-vessel strokes, acute strokes in territories other than the MCA, or who did not undergo a follow-up MRI with T2 and DWI/ADC sequences were excluded from the study. Brain edema and infarct volumes were calculated from manually traced ROIs of the T2 and DWI/ADC images, respectively. Brain regions of interest (ROI) were analyzed using CARESTREAM Vue PACS software (Carestream Health Inc, Rochester, NY). ROIs were manually segmented, and areas within each slice were automatically calculated, summed across the slices, and multiplied by slice thickness to calculate total volume.

### 2.3. Flow cytometric analysis of human monocytes

Peripheral blood samples were collected at 6, 12, and 24 h after initial presentation, then daily for up to seven days by venipuncture or indwelling venous or arterial line. Leukocytes were isolated from whole blood samples by Ficoll (Sigma-Aldrich) density gradient centrifugation. Immune cell isolates were washed and resuspended in phosphate buffered saline (PBS) and stained for CD3, CD45, CD11b, CD19, CD15, CD14, CD16, CD11c, CD8, and PD-1. Data were acquired using a FACSAria (BD) and analyzed using FlowJo (Treestar) (Table S1). The gating strategy is shown in Suppl. Fig. 1.

### 2.4. Mice

Male C57BL/6J (8–10 weeks) wild-type mice (Jackson ME) were maintained at the Johns Hopkins University. Animals were housed in a specific-pathogen-free (SPF) vivarium, which was temperature- and humidity-controlled (21 ± 3 °C, 50 ± 10 %), under a 12 h light/dark cycle. All animals had access to the same food and water *ad libitum* throughout the entire study. Male 8–10 week-old mice were used for all the described studies. Animals were euthanized according to humane endpoints, including central nervous system disturbances, hunched posture, lethargy, weight loss, and inability to ambulate. C57BL/6J wild-type (WT) mice were purchased from The Jackson Laboratory (Bar Harbor, Maine). PD-1−/− (B6.Cg-Pdcd1tm1.1Shr/J) mice were purchased from The Jackson Laboratory (Bar Harbor, Maine). The mice were bred at the Johns Hopkins University Animal Facility per IACUC approved protocol. PD-1^f/fLysMcre^ mice were generated by mating Pdcd1^flox/flox^ (PD-1f/f) mice on a C57BL/6 background with LysMcre mice (B6.129P2- Lyz2tm1(cre)Ifo/J) or CD4cre mice (B6.Cg-Tg(Cd4-cre)1Cwi/BfluJ), all of which were kindly provided by Dr. Vassiliki Boussiotis, Harvard Medical School (Boston, MA) (Strauss et al., 2020). Genetic ablation was confirmed by genotyping of pups through Transnetyx (Memphis, TN).

### 2.5. Transient MCAO

Procedures on male mice were like those previously described (Chiang et al., 2011). Mice were induced and maintained with 3 % and 1.5 % isoflurane, respectively, in enriched O2 using a vaporizer. Rectal temperature was maintained at ~37 °C. The mouse was placed prone and a right-sided scalp incision was made midway between the lateral canthus and the anterior pinna. A laser Doppler (Perimed PF 6000) flow probe was attached perpendicularly to the right lateral parietal skull. The mouse was then positioned supine. A midline cervical incision was made under an operating microscope, and under high magnification, the right external carotid artery (ECA), right common carotid artery (CCA) and right internal carotid artery (ICA) were dissected free of the surrounding tissue. The ECA was permanently ligated with a 7–0 silk suture, and the ICA and CCA were occluded with 3 mm temporary aneurysm clips (Aesculap). A silicone-coated 6–0 nylon suture (Doccol, 602223PK10Re) was passed into the lumen of the ECA and guided into the ICA. The temporary clip on the ICA was removed and the suture was advanced past the skull base until resistance was met. MCA territory flow occlusion was verified by a sustained 80 % drop of baseline cortical perfusion values. After 45 min of occlusion, the suture was withdrawn to allow reperfusion of the vessel and the incisions were closed. Any animal that experienced technical complications during the induction of MCAO, such as excessive bleeding, prolonged operation time, or a less than 80 % drop of baseline cortical perfusion values as measured by laser Doppler were excluded. Survival rates were documented daily for up to 30 days after surgery. Sham-operated mice underwent the same surgical treatment, with all the arteries exposed and the ECA ligated, but without filament insertion into the MCA.

### 2.6. PD-L1 administration

Recombinant Mouse B7-H1 (PD-L1, CD274)-Fc Chimera (carrier-free) (Biolegend, Cat 758208) was diluted in sterile PBS such that 50 μg of PD-L1 in a total volume of 200 mL was administered by intraperitoneal injection 1 h and 24 h after reperfusion.

### 2.7. Brain water content

Mice were deeply anesthetized and sacrificed by decapitation at 48 h after MCAO, after which the brain was quickly removed and heated for 72 h at 100 °C in a drying oven (Vacutherm, Fisher Scientific). Brain water content was measured by comparing wet-to-dry ratios as previously described (Keep et al., 2012). Tissues were weighed with a scale to within 0.001 mg. Tissue water content was then calculated as (1) Water content = (wet weight - dry weight)/dry weight, and (2) %Water content = 100x(wet weight - dry weight)/wet weight.

### 2.8. MRI acquisition and analysis

High-resolution MRI was performed 72 h after MCAO surgery on an 11.7 T Ultra Shielded Bruker Biospec system (Bruker, Ettlingen, Germany) with a horizontal bore. Images were acquired using a 72-mm quadrature volume resonator as a transmitter, and a four-element (2 × 2) phased-array mouse-head coil as a receiver. The animal was fixed on a plastic holder and anesthetized with 3–4 % isoflurane for induction, and 1–2 % for maintenance. The respiratory rate was monitored using a pressure pad and a thermostatically controlled heating reel was used to keep the body temperature at 37 degrees. Details on acquisition protocols and processing of MRI are provided in the data supplement. Apparent diffusion coefficient (ADC), spin echo echo planar imaging diffusion-weight imaging (SE-EPI-DWI), and T2 MRI sequences were acquired.

Brain regions of interest (ROI) were analyzed using Paravision 6.01 software (Bruker, Ettlingen, Germany). Brain edema and infarct volumes were calculated from manually traced ROIs of the T2 and DWI/ADC images, respectively. Areas within each slice were calculated, summed across the slices, and multiplied by slice thickness to calculate total volume.

### 2.9. Immune cell isolation

To isolate brain-infiltrating immune cells, brains were harvested 48 h post-MCAO. The brains were mechanically homogenized and filtered on ice, then resuspended in 5 mL 70 % Percoll, layered below 7 mL of 37 % Percoll and centrifuged at 2000 rpm for 20 mins at room temperature. The cell layer at the 37 %/70 % interface was collected and washed with PBS.

### 2.10. Flow cytometric analysis of murine monocytes

To analyze surface markers, immune cells were pre-treated with Fc Block (anti-CD16/32) (BD Biosciences), washed, and stained with Live-Dead Aqua (Invitrogen) at 4 °C for 30 min. Cells were then washed and stained at 4 °C for 30 min with CD45, CD11b, CD3, CD8, Ly6C, Ly6G, CCR2, and PD-1 (Table S5 for antibody clones and dilutions). Data were acquired using a FACSAria (BD) flow cytometer and analyzed using FlowJo software (TreeStar). Nonviable cells were excluded by forward versus side scatter analysis and Live-Dead Aqua (Invitrogen) staining. The gating strategy is shown in Suppl. Fig. 2.

### 2.11. Behavioral tests

Functional outcomes were assessed using a 28-point neuroscore at 1, 2, 3, and 4 weeks after surgery by a blinded, experienced experimenter. Mouse gait was assessed using a motorized treadmill (DigiGait Imaging System, Mouse Specifics, Boston, MA) and analyzed using DigiGait analysis software (Mouse Specifics, Inc.). Exploratory and locomotor activity of mice was measured by open field test at 4 weeks post-surgery. Behavioral tests were performed in a well-lit environment. Animals were acclimated in the testing room for an hour before testing, and the testing areas were thoroughly cleaned between animals.

#### 2.11.1. 28-point neuroscore

Functional deficits were assessed with the 28-point neuroscore test as described previously (Encarnacion et al., 2011). The cumulative maximum score of 28 point is based on eleven individual tests: (1) circling behavior (maximum 4 points), (2) motility (maximum 3 points), (3) general condition (maximum 3 points), (4) paw placement (maximum 4 points), (5) righting reflex (maximum 1 point), (6) pullingup on a horizontal bar (maximum 3 points), (7) climbing on an inclined plane (maximum 3 points), (8) grip strength (maximum 2 points), (9) contralateral reflex (maximum 1 point), (10) forepaw reaching (maximum 2 points), and (11) contralateral rotation when held by the tail (maximum 2 points). Scoring was determined on a scale from 0 (worst functional and behavioral outcome) to the maximum score of 28 points for healthy animals (Encarnacion et al., 2011; Arkelius et al., 2020).

#### 2.11.2. Open field

The open field test was used to assess locomotor activity and exploration habits of mice in a relatively large, circular enclosure. A circular open field arena (40 cm diameter) was created using a white PVC cylinder (0.41 m diameter, 0.3 m height) and placed in a quiet, well-lit room (130 lm). The open field was divided into three concentric circles—center, neutral, and peripheral zones. Each mouse was placed against the wall of the arena and allowed to explore freely for 15 min. Anymaze^™^ video-tracking software was used to record and measure behavior as described previously (Jantzie et al., 2018).

#### 2.11.3. Gait assessment

Gait analysis was performed using the DigiGait Imaging System (Mouse Specifics Inc., Boston, MA) as previously described (Vincelette et al., 2007). Digital video images of the animal’s underside were collected with a high-speed video camera placed below the transparent belt of a motorized treadmill (Jantzie et al., 2014; Koshimizu et al., 2014). All settings, such as the camera focus, lighting, and belt speed, were optimized before testing. Mice were forced to walk or run at a fixed velocity (Yang et al., 2022), which was preset to 10 cm/s at week 1 and 15 cm/s at week 3. The mice were acclimated to the enclosure and the walking speed before recording for analysis. DigiGait analysis software v.12.2 (Mouse Specifics, Inc.) identified each paw as the mouse walked on the belt. Only video recordings in which a mouse walked without stumbling and did not contact the walls or bumpers were used for measurements and analyses as established in literature. Poor quality video or gait analysis tracings were excluded. The areas of each paw relative to the belt and camera were calculated throughout each stride. When plotted as a function of time, the areas provided a dynamic gait signal that denoted the braking, swing, and propulsion components of each stride. Stride, stance, and swing durations were also calculated from these signals. As previously described, additional variables were calculated by the software to describe other aspects of gait, including paw angle and relative positions of the paws to each other and to the midline (Vincelette et al., 2007; Berryman et al., 2009).

### 2.12. Single cell RNA sequencing and data analysis

Blood was collected by cardiac puncture from anesthetized mice 48 h after MCAO. The blood was centrifuged at 300G for 5 min and resuspended in ACK lysing buffer (Life) three times for five minutes on ice to lyse nucleated red blood cells (RBCs). To account for biologic variations, cells from 10 mice that underwent MCAO in the same surgery session were pooled for each sample: (1) MCAO and (2) MCAO + PD-L1. The cells were then washed with PBS and stained with CD3, CD45, CD11b, and PD-1 (Suppl. Table 1). Dead cells were excluded with propidium iodide (PI) staining. PD-1+ versus PD-1− monocytes (CD45 + CD11b + CD3−) were gated and sorted into sterile RPMI (Gibco) supplemented with 5 % FBS (Fisher). Methods for Single-Cell RNA-Sequencing using the 10x Genomics Platform are described in the Supplementary materials.

Differential gene expression, geneset enrichment analysis, clustering, and visualization of the single-cell RNA sequencing data was performed in R (version 4.1.0) as follows:

The cell-by-gene count matrix was filtered to exclude cells with fewer than 1000 total RNA counts across all genes and to exclude genes with fewer than 10 counts across all cells. The resulting cell-by-gene matrix contained 15,396 genes and 27,431 cells. Counts were then normalized using library size normalization and scaled by 1e6.

To test for differentially expressed genes between PD-L1 treated and untreated monocytes, cells from the PD-1 positive and negative compartments for each treatment condition were combined. Differentially expressed genes were determined using a two-sided Wilcoxon rank sum test with a Benjamini-Hochberg multiple hypothesis correction and a p-value cutoff of 0.05. Log fold changes in gene expression between PD-L1 treated and untreated monocytes were computed as the log_2 (mean gene expression in treated cells/mean gene expression in untreated cells), where gene expression is the library size normalized and scaled counts.

Gene set enrichment analysis (Subramanian et al., 2005) was performed using the fgsea package (version 1.20.0) (Korotkevich et al., 2016). Significantly differentially expressed genes were ranked by decreasing log fold change. GSEA was performed with the Gene Ontology Biological Processes gene sets (Liberzon et al., 2011), with significantly enriched genesets determined using an adjusted p-value cutoff of 0.05.

To obtain cell clusters, the CPM-normalized count matrix was first log_10 transformed with a pseudocount of 1. Principal components analysis (PCA) was performed on the centered and unit scaled normalized count matrix using the svds function in the package RSpectra (version 0.16) with 50 principal components. A nearest neighbor graph with parameter k = 100 was constructed using the nn2 function in the package RANN (version 2.6.1) and clusters were obtained using the cluster_louvain function in the package igraph (version 1.2.11). To test if the proportion of treated cells in each cluster was significantly different from the proportion of treated cells in the overall data, the prop.test function in the package stats in base R was used.

The data were visualized in a two-dimensional uniform manifold approximation and projection embedding constructed using the umap function in the package uwot (version 0.1.11) using the 50 principal components and parameters n_neighbors = 500, and min_dist = 1, with other parameters with default values.

### 2.13. Statistical analyses

#### 2.13.1. Murine data

Survival was analyzed by Kaplan-Meier method and compared by log-rank Mantel Cox test. Unpaired non-parametric Mann-Whitney tests were used to make comparisons between two groups. Comparisons within groups were presented as mean ± SEM. All murine data excluding the behavior tests were analyzed using GraphPad Prism 9 and values of p < 0.05 were considered significant.

#### 2.13.2. 28 Point neuroscore

The 28 point neuroscores at week 1 for treated and untreated wild-type mice were categorized by quartile (1st, 2nd, 3rd,4th), such that the 4th quartile represented the mice scoring in the top 25th percentile on the 28 point neuroscore. Subsequently, the Chi-squared and Fisher exact tests were used to evaluate the association between treatment with PD-L1 and having a high 28 point neuroscore (falling in the 4th quartile vs falling in any one of the 1st three quartiles). The same process (categorization followed by Chi-squared and Fisher exact testing) was implemented for global PD-1 KO and then myeloid-specific PD-1 KO mice.

#### 2.13.3. DigiGait and open field

Scores on the various variables of digigait at week 1 and week 3 were compared between sham, treated stroke, and untreated stroke mice using the Kruskal-Wallis test. Post-hoc comparisons with Bonferroni correction were subsequently performed for variables with significant overall Kruskal-Wallis p-values. To provide a more global picture of the effect of PD-L1 on variables that are significantly altered by stroke compared to sham, the results for these variables were plotted in the form of horizontal bar graphs delineating the percent change in median compared to sham in treated versus untreated mice. A similar process was used to analyze the open field variables at week 4.

#### 2.13.4. Human patient data

Baseline characteristics of enrolled patients were summarized using descriptive statistics (n,% for categorical variables and mean ± SD with median, IQR for continuous variables). Regarding flow cytometry data, the mean fluroescence intensity (MFI) values for PD-1 fluorescence on different monocyte cell populations (total, classical, intermediate, nonclassical) were plotted at different time points. We assessed maximum values and daily average values. Since some patients had several values collected at different time-points within the first 24-hour period since time last known well, the maximum value for the first 24 h was selected as the “day 1” value for the daily average calculations. Nonparametric analyses using Spearman’s correlation coefficient were performed to evaluate correlations between the MFI values for PD-1 fluorescence on monocyte cell subpopulations and edema-to-infarct ratios on MRI. Further analyses investigating potential linear associations were conducted using Pearson’s correlation coefficient while acknowledging limitations of small sample size and the presence of outliers. Statistical significance was set at p < 0.05 with 2-sided hypothesis testing, and analyses were performed using SPSS software (version 25.0; SPSS Inc, IBM, Armonk, New York).

##### 2.13.4.1. In vitro polarization of myeloid cells.

Hematopoietic stem cells were isolated from the femur and tibia of C57BL/6 mice and treated with ACK lysing buffer for red blood cell lysis. Cells were plated with myeloid-polarizing cytokines- murine M–CSF (40 ng/ml) and GM-CSF (40 ng/ml), for 2 days. Cells were plated on control or PD-L1-immobilized plates (pre-coated with 1ug/ml of soluble PD-L1 overnight) and with isotype control or anti-PD-1 (1ug/ml or 10ug/ml doses). On day 3, flow cytometry was conducted to assess monocyte polarization.

##### 2.13.4.2. Seahorse assay for measurement of mitochondrial respiration.

Hematopoietic stem cells were isolated from the femur and tibia of C57BL/6 mice and treated with ACK lysing buffer for red blood cell lysis. Cells were plated with myeloid-polarizing cytokines, murine M–CSF (40 ng/ml) and GM-CSF (40 ng/ml), for 2 days. Cells were plated on control or PD-L1-immobilized plates (pre-coated with 1ug/ml of soluble PD-L1 overnight) and with isotype control or anti-PD-1 (1ug/ml or 10ug/ml doses). On the third day, polarization to myeloid cells was confirmed by flow cytometry. Mitostress test was performed with the polarized cells using the Seahorse XF Pro Analyzer (Agilent Technologies, Massachusetts, USA). Prior to seeding on Seahorse cell culture microplates, myeloid cells were washed with buffered Seahorse XF Base Medium. After washing the cells, 200,000 cells were transferred to the microplates and incubated with buffered Seahorse XF Base Medium supplemented with 2 mM glutamate, 1 mM sodium pyruvate and 10 mM glucose for an hour at 37 °C in a non-CO_2_ incubator. Oxygen consumption rate (OCR) was measured under basal conditions and in response to 1.5 μM Oligomycin, 1 μM fluoro-carbonyl cyanide phenylhydrazone (FCCP), and 100 nM rotenone + 1 μM antimycin A.

## 3. Results

### 3.1. PD-1 was expressed on monocytes and correlated with radiographic edema in LVO patients

**Fig. 1.**
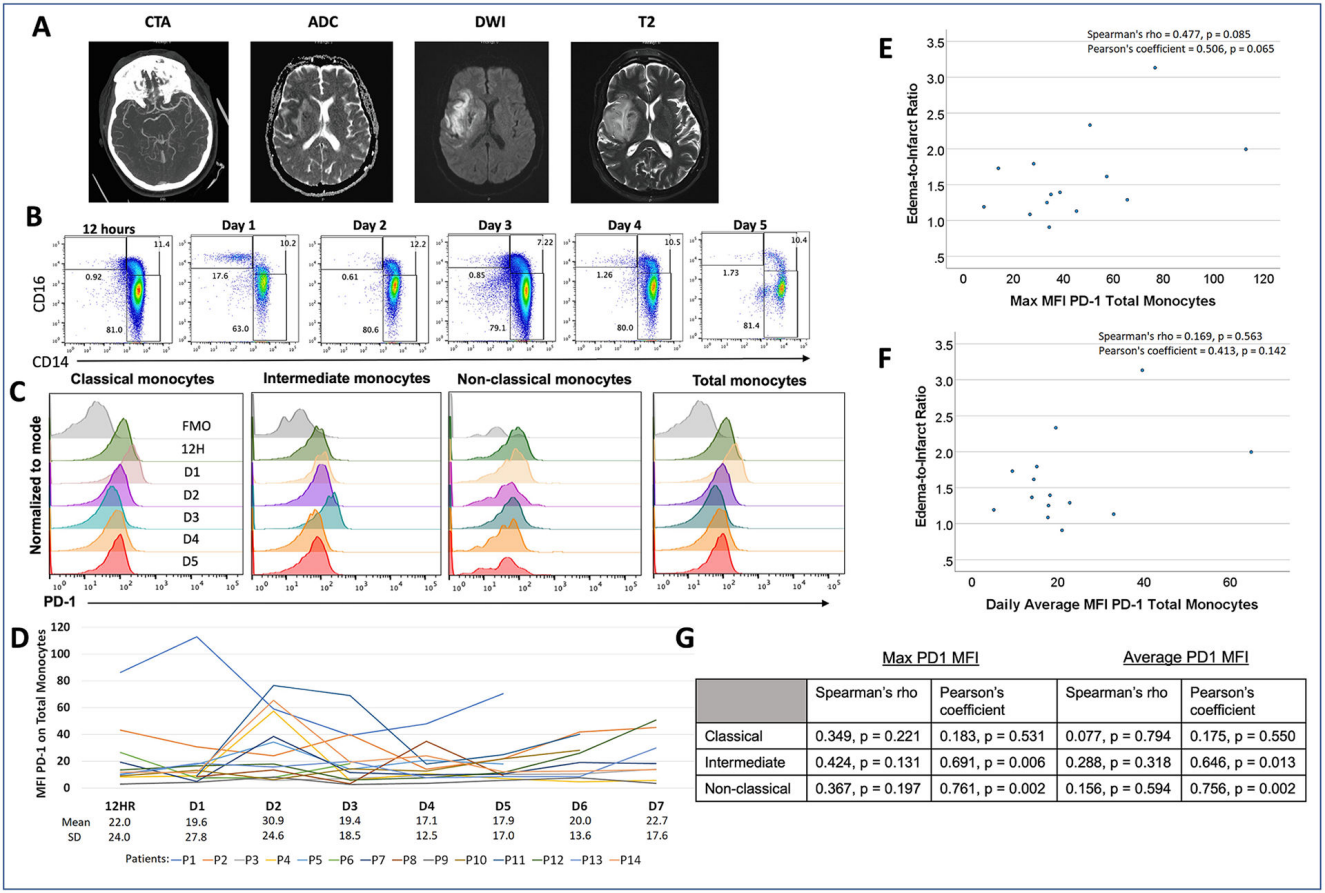
PD-1 was upregulated on circulating monocytes after stroke. (**A**) Representative patient images of an acute right MCA occlusion on CTA, and associated MRI images demonstrating a large MCA territory infarct (ADC/DWI) with associated edema (T2). (**B**) Flow cytometric analysis showing frequencies of monocyte subsets in the blood during the acute period after MCA occlusion and reperfusion. (**C**) Changes in PD-1 expression on blood monocytes after MCA occlusion and reperfusion. (**D**) PD-1 mean fluorescence intensity (MFI) on blood monocytes after MCA occlusion and reperfusion in a cohort of 14 patients. (**E, F**) Correlation analysis of maximum and average PD-1 MFI on circulating monocytes with edema-to-infarct ratio. (**G**) Correlation analysis of maximum and average PD-1 MFI on monocyte subsets with edema-to infarct ratio.

To determine if circulating PD-1+ monocytes correlate with neurologic outcomes in humans, we studied 14 patients who presented to our institution with an LVO of the right or left MCA. CT angiography (CTA) showing a right MCA occlusion and MRI sequences showing stroke volume (ADC and DWI) and surrounding edema (T2) from a representative case are presented in Fig. 1A. The average age was 62 years, and the most frequently affected vessel was the M1 segment (most proximal) of the left MCA. Baseline and imaging outcome characteristics are presented in Table 1. A total of 12 patients (86 %) underwent mechanical thrombectomy, and the mean edema-to-infarct ratio on magnetic resonance imaging (MRI) was 1.6. PD-1 expression was observed in classical (CD14^hi^, CD16^lo^), intermediate (CD14^hi^, CD16^hi^), and non-classical (CD14^lo^, CD16^hi^) monocyte populations (Fig. 1B). The pattern of PD-1 staining on monocytes was a shift rather than the well-delineated PD-1+ population observed on lymphocytes. This pattern is consistent with previous reports of PD-1 staining on myeloid cells (Strauss et al., 2020; Christofides et al., 2023; Gordon et al., 2017). The highest PD-1 fluorescence on total monocytes was observed on day 2 (30.9 ± 24.6); however, there was considerable inter- and intra-patient variability in timing and degree of PD-1 expression on monocytes as demonstrated by the individual patient graphs in Fig. 1C. Higher daily average (Fig. 1D) and maximum PD-1 MFI values on total monocytes (Fig. 1E) correlated with increased edema-to-infarct ratios graphically, with trends towards significance noted for max values (Spearman’s rho = 0.477, p = 0.085, Pearson’s correlation coefficient = 0.506, p = 0.065). Significant correlations between MFI for PD-1 and edema-to-infarct ratio were noted in our Pearson correlation analysis in the intermediate and nonclassical monocyte subtypes.

### 3.2. Administration of soluble PD-L1 reduced cerebral edema and improved survival in mice

**Fig. 2.**
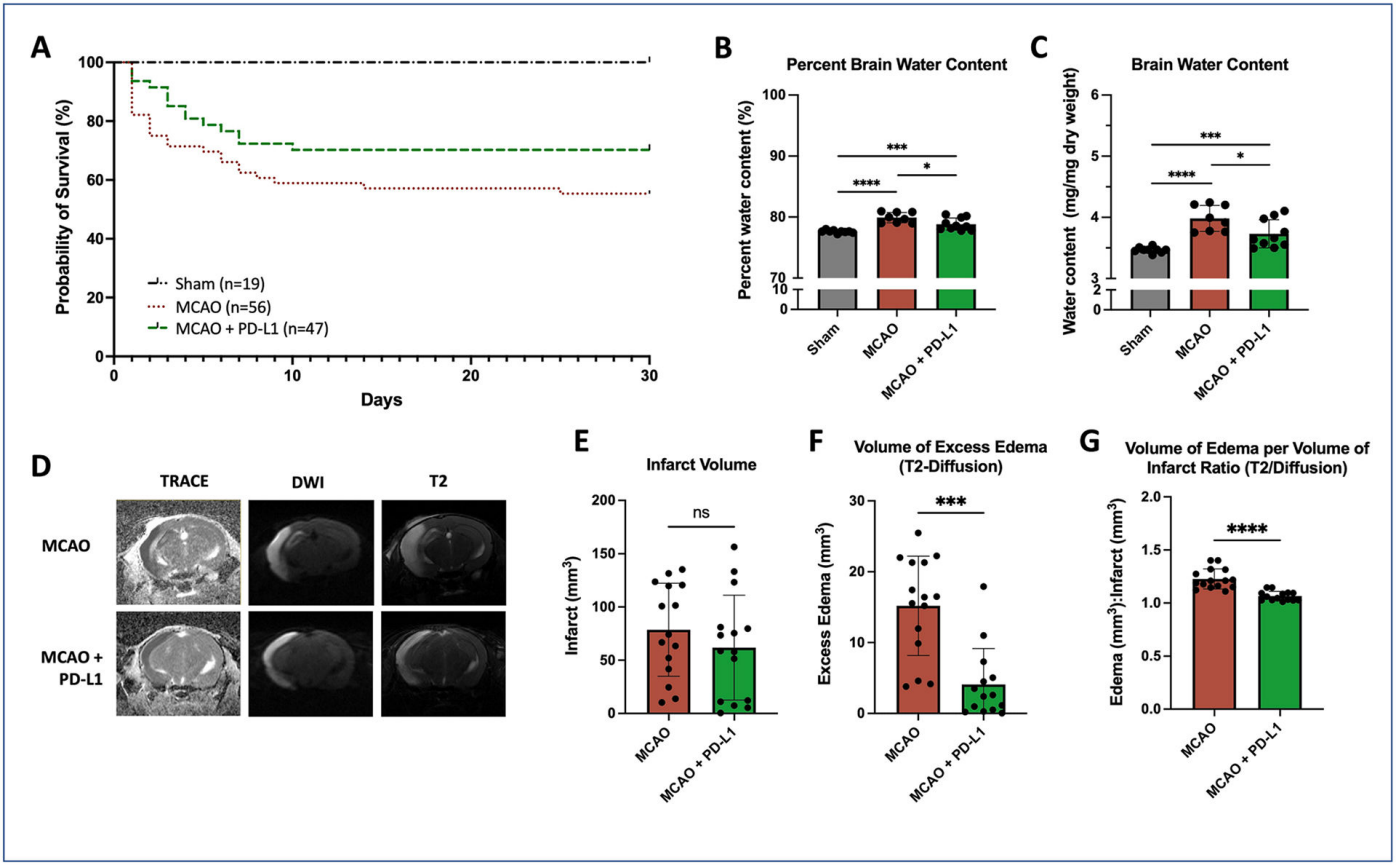
PD-L1 attenuated brain injury and improved survival after MCAO. (**A**) Kaplan-Meier curve. 30-day survival rate after MCAO was significantly increased by treatment with soluble PD-L1. P < 0.0022, log-rank test; n = 19 to 56 mice per experimental arm. (**B, C**) Bar graphs demonstrate that brain water content is increased at 48 h after MCAO. Treatment decreases water content, though not back to baseline. P < 0.0001 sham versus MCAO; p = 0.0003 sham versus MCAO + PD-L1; p = 0.0266 MCAO versus MCAO + PD-L1N = 8–10 mice per experimental group. (**D**). Representative MRI images of wild type mice, 72 h after MCAO. ADC and TRACE sequences show a right MCA territory infarct pattern. The T2- weighted sequence demonstrates brain edema (high signal intensity) within and surrounding the core infarct. (**E**) Bar graph demonstrates no significant change in infarct volume with treatment (p = 0.3295). However, treatment significantly decreases the volume of edema surrounding the core infarct (**F**), p = 0.0001, and volume of total edema per volume of core infarct (**G**), p < 0.0001. n = 14 to 15 per experimental group. Statistical analyses were performed by Mann-Whitney test. Error bars represent +/− SEM.

We evaluated the effects of IP injection of sPD-L1 (administered at 1 h and 24 h after ischemic insult) on functional and radiographic outcomes in a transient MCAO model. Administration of sPD-L1 improved overall survival, particularly in the first 48 h after infarct (Fig. 2A). The overall survival rate for the untreated MCAO mice was 55.36 % (n = 56), versus 70.21 % for MCAO mice that received sPD-L1 (n = 47; χ^2^ = 12.23, p = 0.0022).

Because the difference in survival was most evident in the acute phase, we hypothesized that treatment with sPD-L1 decreased early brain swelling. Brain water content was measured at 48 h post-MCAO as previously described (Fig. 2B,C) (Keep et al., 2012). Compared to animals that underwent sham surgery, mice undergoing MCAO without treatment had a 2.28 % increase in whole brain water content (77.61 % and 79.89 %, respectively, p=<0.0001).

Treatment with sPD-L1 significantly decreased the percent water content to 78.82 % (p = 0.0266).

To quantify the relationship between infarct and edema volumes, high resolution T2, DWI, and ADC MRI sequences were obtained at 72 h post-MCAO (Fig. 2D). Total infarct volume, as visualized by areas of restricted diffusion on both DWI and ADC maps, was not significantly changed by sPD-L1 treatment (Fig. 2E). However, treated mice had significantly lower volumes of excess edema, as defined by total volume of T2 hyperintensity subtracted by the total volume of diffusion restriction (p < 0.0001) (Fig. 2F). This relationship remained significant when normalizing for infarct volume (p < 0.0001) (Fig. 2G).

### 3.3. Soluble PD-L1 improved early performance scores in mice with potential for good functional recovery, but did not improve early recovery in severely injured mice

**Fig. 3.**
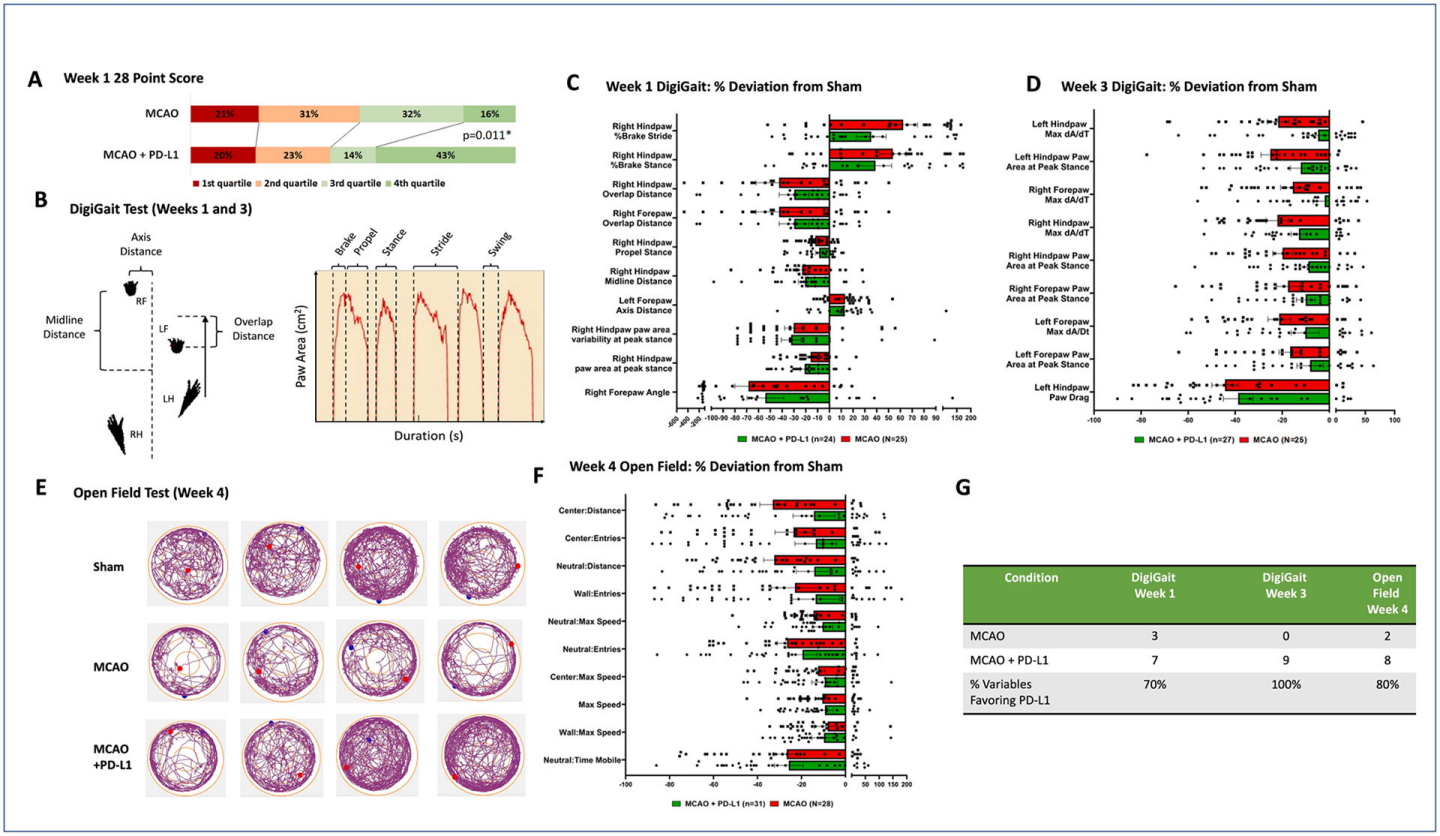
Soluble PD-L1 therapy improved short- and long-term functional outcomes. (**A**) Bar chart shows the 28-point neuroscores at week 1 for wild-type mice categorized by quartile (1st, 2nd, 3rd, 4th). Statistical analyses were performed by Chi-square and Fisher exact tests to evaluate the association between treatment with PD-L1 and having a high 28-point neuroscore (4th quartile). P = 0.011, n = 35 to 38 per experimental group. (**B**) Graphical representation of select gait parameters (**C, D**) Bar graphs depict percent change in median when compared to sham mice. Displayed are all the variables that were significantly modified by stroke for the Digigait test at week 1 and week 3. (**E**) Representative tracings of the mouse’s trajectory in an open field. (**C, D**) Bar graphs depict percent change in median when compared to sham mice. Displayed are all the variables that were significantly modified by stroke for the Digigait test at week 1 (**C**) and week 3 (**E**), as well as the open field test at week 4 (**F**). Significant p < 0.05 when comparing MCAO and sham after a Bonferroni correction for multiple comparisons. (**G**) Table summarizes the number of variables that, with treatment, more closely resembled sham animals than untreated MCAO animals.

To evaluate functional outcomes, the 28-point neuroscore—a well-established sensorimotor behavior test (Encarnacion et al., 2011)—was administered weekly after MCAO. Like humans, mice have a range of outcomes after stroke. To account for this variation the scores were broken down into quartiles to determine if subgroups of mice were affected by treatment (i.e. categorized by 1st, 2nd, 3rd and 4th quartiles such that the 4th quartile represented the mice scoring in the top 25th percentile on the 28 point neuroscore). Animals treated with sPD-L1 were significantly more likely to fall into the highest quartile (71 %, p = 0.011) (Fig. 3A). Based on raw scores alone, there was no significant difference between the untreated and treated cohorts (Suppl. Fig. 3). By weeks 2, 3, and 4, a difference was no longer discernable (Table S1-3), consistent with previous reports of good spontaneous recovery after MCAO as measured by 28-point neuroscore (Encarnacion et al., 2011). These findings indicate that mice with a higher potential for functional recovery benefit from sPD-L1, while the effects of immune activation are overshadowed by the primary ischemic injury in poorly performing mice. sPD-L1 in sham operated mice did not affect 28-point neuroscore or immune phenotype of circulating or brain infiltrating myeloid cells (Suppl. Fig. 4).

### 3.4. PD-L1 treated mice more closely resembled sham operated animals on assessments of long-term functional recovery

To assess long-term functional outcomes, locomotor function was assessed using the Digigait system (Fig. 3B). Significance was determined by a p value < 0.05 after a Bonferroni correction for multiple comparisons (Table 2,3). At 1-week post-MCAO, computerized treadmill gait analysis of the sham surgery versus the untreated control cohorts revealed 10 gait parameters that were significantly altered by stroke. In the sPD-L1 treatment cohort, 7 of these 10 parameters were closer to sham than the untreated stroke group (Fig. 3C). At week three, all parameters (9/9) that were significantly affected by stroke showed a trend towards improvement with PD-L1 treatment (Fig. 3D). The open field test was administered at week four to evaluate overall activity levels, locomotor ability, exploration habits, and anxiety (Fig. 3E). Comparison of the sham versus untreated animals revealed ten parameters that were significantly altered by stroke. Eight of these variables trended closer to sham animals in the PD-L1 treated group (Fig. 3F). Due to high variance in these interconnected behavioral measures, none of the individual variables reached significance for treated vs. untreated stroke mice (Tables 2, 3, 4).

### 3.5. PD-1 was upregulated on infiltrating brain myeloid cells

**Fig. 4.**
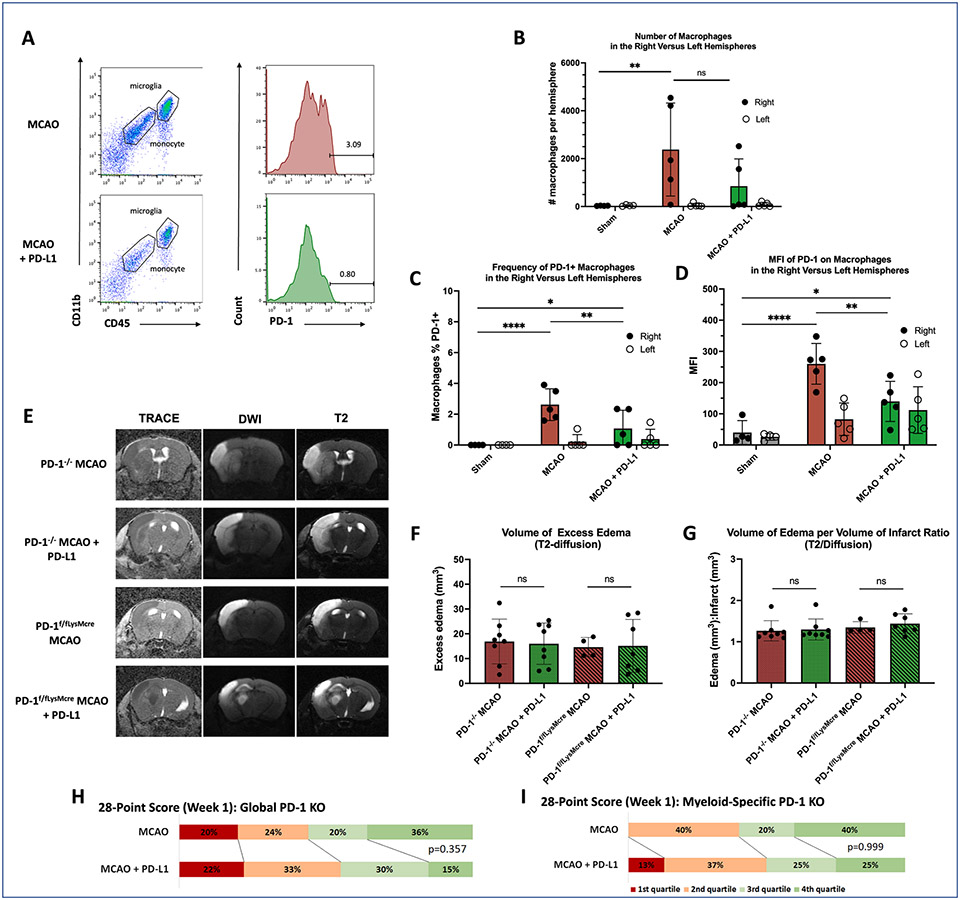
Brain infiltrating myeloid cells were critical mediators of the sPD-L1 treatment effect. (**A**) Representative flow cytometry plots to illustrate gating strategy for PD-1 + monocytes (CD11b + CD45^hi^). (**B**) MCAO results in significant infiltration of monocytes into the ischemic hemisphere (p = 0.0159) at 48 h after MCAO. Though the total numbers are not significantly affected by treatment, PD-L1 administration significantly decreases the frequency of PD-1 + monocytes (**C**) and overall expression as measured by mean fluorescence intensity (MFI) (**D**). Experiments run in duplicate with n ≥ 4 mice per arm. (**E**) Representative MRI images of global myeloid knockout (PD-1^−/−^) mice and myeloid-specific knockout (PD-1^f/fLysMcre^) mice, 72 h after MCAO. ADC (low signal intensity) and TRACE (high signal intensity) sequences depict the core infarct, while the T2- weighted (high signal intensity) sequence demonstrates edema. (**F**) Bar graph demonstrates no significant change in the volume of edema surrounding the core infarct or for the volume of total edema per volume of core infarct (**G**). N = 8 per group for the global PD-1 knockout arms, and n = 4 to 7 for the myeloid-specific knockout arms. Statistical analyses were performed by Mann-Whitney test. Error bars represent +/− SEM. (**H-I**) Bar chart depicts the 28-point neuroscores at week 1 for global and tissue-specific PD-1 knockout mice categorized by quartiles. Statistical analyses using Chi-square and Fisher exact tests demonstrated no significant association between PD-L1 treatment and having a high 28-point neuroscore (4th quartile). N = 25 to 27 per group for the global PD-1 knockout arms, and n = 4 to 8 for the myeloid-specific PD-1 knockout arms.

To determine whether PD-1 + infiltrating monocytes were present in the ischemic hemisphere after stroke, we isolated immune cells from the left and right hemispheres 48 h after MCAO (Fig. 4A). Comparison of the right versus left hemispheres revealed a significantly higher number of CD11b+, CD45^hi^ monocytes in the ischemic hemisphere compared to the non-ischemic hemisphere (Fig. 4B). sPD-L1 treatment resulted in a lower overall frequency of PD-1 positivity amongst the infiltrating macrophage population (p = 0.0071) (Fig. 4C) by one-way ANOVA and Holm-Sidak’s tests for multiple comparisons, and a trend towards decreased PD-1 expression as measured by MFI (p = 0.0059) (Fig. 4D), but did not decrease the overall number of monocytes. There was no significant change in CCR2 expression on infiltrating monocytes (data not shown).

### 3.6. Myeloid-specific knockout of PD-1 abrogated the PD-L1 treatment effect

To determine if PD-1 + myeloid cells were the targets of sPD-L1, MCAO was performed on two sets of age-matched transgenic mice: (1) global PD-1 knockout (PD-1^−/−^) and (2) myeloid-specific PD-1 knockout mice (PD-1^f/fLysMcre^). High-resolution MRI was performed at 72 h to determine if loss of PD-1 expression would abrogate the treatment effect (Fig. 4E). Volumetric analysis revealed that sPD-L1 treatment had no effect on total infarct volume, excess edema volume, or excess edema per infarct volume in both the PD-1^−/−^ and PD-1^f/fLysMcre^ mice (Fig. 4F, G). The 28-point neuroscore at week 1 was not affected by sPD-L1 administration in either the PD-1^−/−^ or PD-1^f/fLysMcre^_m_ice (Fig. 4H, I). By both metrics, the knockout mice resembled untreated mice, indicating worse outcomes after MCAO when PD-1 was ablated in myeloid cells, either in a global or tissue-specific manner.

### 3.7. Soluble PD-L1 treatment reprograms circulating monocytes

**Fig. 5.**
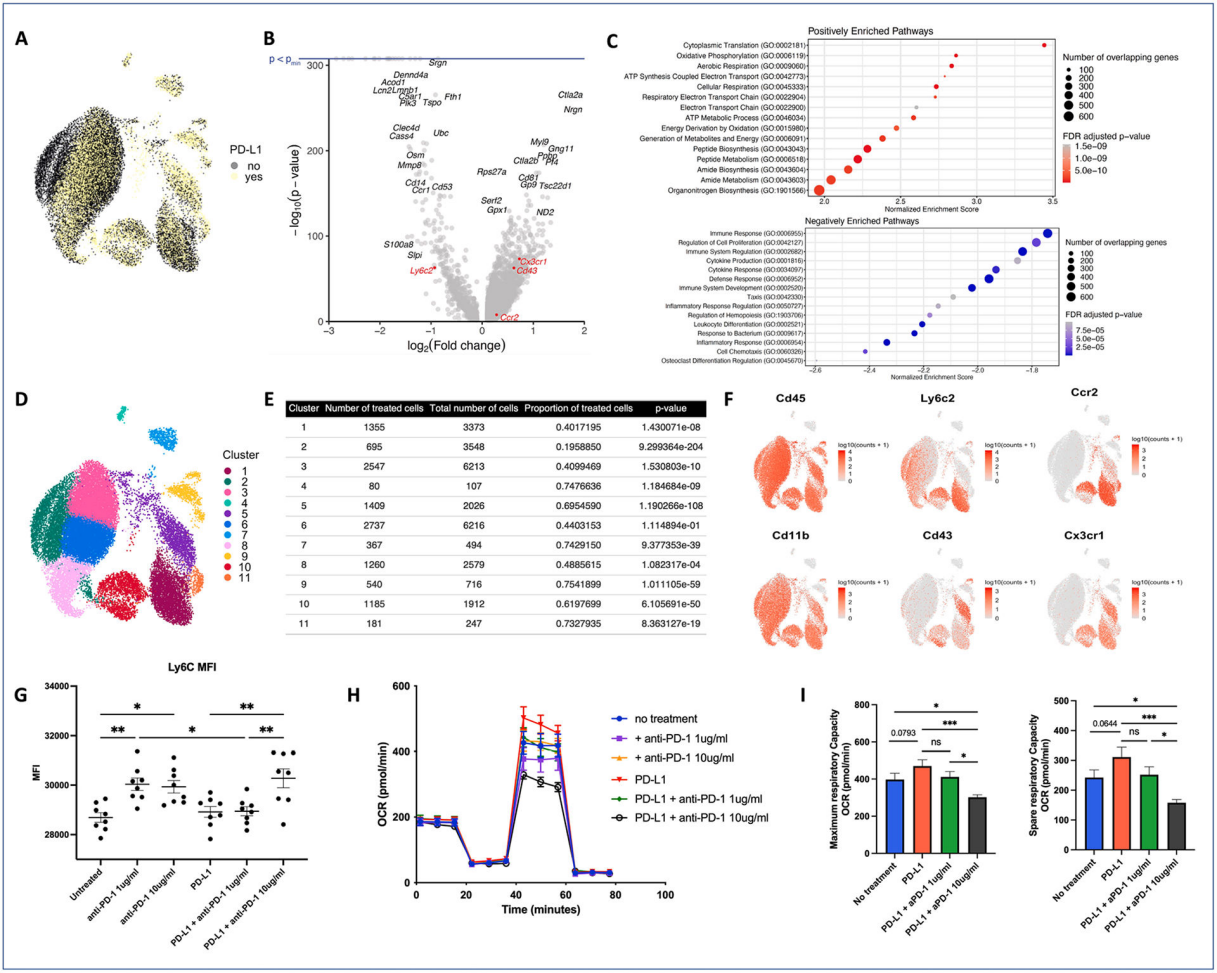
Soluble PD-L1 reprogrammed circulating monocytes to a restorative subtype with downregulation of inflammatory markers and increased metabolic reserve. (**A**) UMAP of PD-L1 treated and untreated monocytes single cell RNA sequencing demonstrated the transcriptional heterogeneity of PD-L1 treated and untreated murine monocytes isolated at 48 h after MCAO. (**B**) Volcano plot showing 5,455 significantly differentially expressed genes (Benjamini-Hochberg adjusted p < 0.05). Horizontal blue line indicates pmin, which is the smallest p-value represented in R (~2.23 e-308). Points at the blue line indicate genes with p-value < pmin. (**C**) Top 15 positively (red) and negatively (blue) enriched gene sets from Gene Ontology: Biological Processes. (**D**) UMAP showing 11 transcriptional clusters derived from Louvain community detection. (**E**) Table demonstrating number of PD-L1 treated cells, total cells and proportion of treated cells in each cluster and p-value of differential proportion test between proportion of PD-L1 treated cells in each cluster and proportion of PD-L1 treated cells in the overall data. (**F**) UMAP showing expression of select genes for monocyte subset and activation markers. (**G**) Scatter plot showing the effects of exposing *in vitro* myeloid cells to anti-PD-1 antibody and/or sPD-L1 on Ly6C expression. (**H**) Oxygen consumption rate of myeloid cells treated with PD-L1 +/− anti-PD-1 blocking antibody (**I**) Bar graphs depict calculated values for respiratory parameters.

Single cell RNA sequencing of all CD45 + CD11b + CD3− immune cells in the blood 48 h after MCAO was performed to characterize circulating monocytes in untreated and sPD-L1 treated mice (Fig. 5A). Differential gene expression analysis performed between the untreated and treated monocytes identified 5,455 differentially expressed genes (DEGs) (Fig. 5B). Gene set enrichment analysis revealed a positive enrichment in metabolism and biosynthesis genesets and a negative enrichment in immune and inflammatory process genesets in monocytes isolated from PD-L1 treated mice (Fig. 5C). From all sequenced cells, 11 clusters were identified (Fig. 5D) and proportion of cells that were derived from sPD-L1 treated mice were compared for each cluster (Fig. 5E). RNA expression of markers associated with monocyte ontogeny and activation status were evaluated to explore potential phenotypes of the monocyte subpopulations (Fig. 5F). To further investigate transcriptional differences between the monocyte subpopulations, differential gene expression between cells in each cluster and all other cells in the data was performed (Data file 2). Most clusters showed robust expression of *CD45* and *CD11b*, indicating limited contamination of other cell types during the sorting process. Clusters 5 and 10, which exhibited lower expression of *Ly6c2* and higher expression of *Cd43*, were significantly enriched in sPD-L1 treated mice. Monocyte clusters 1 and 10 displayed higher expression of *Ly6c* and *Ccr2* (associated with a “classical” pro-inflammatory phenotype) (Rahman et al., 2017; Hammond et al., 2014) and lower expression of *Cd43*, whereas monocyte cluster 5 had higher expression of *Cd43* and lower expression of *Ly6c* and *Ccr2*, indicative of a “non-classical” patrolling phenotype (Thomas et al., 2015) (Fig. 5F). Of note, cluster 5 was also enriched for PD-L1 + monocytes (Suppl. Fig. 5B), which has been reported to be a specific marker of non-classical monocytes (Bianchini et al., 2019). Expression of Cx3cr1, a chemokine receptor that mediates anti-inflammatory, patrolling monocyte activity (Gliem et al., 2016) was more highly expressed in monocytes isolated from treated mice. Thus, treatment with sPD-L1 appears to shift the monocyte subpopulations from a Ly6^hi^, CCR2^hi^, CD43^lo^ to a Ly6C^lo^, CX3CR1^hi^, CD43^hi^, PD-L1 + phenotype.

Given the prominence of metabolic pathways affected by sPD-L1 treatment and to confirm agonist activity of sPD-L1 we studied functional consequences of sPD-L1 and PD-1 blocking antibodies in an *in vitro* myeloid precursor activation assay. We first confirmed that this assay recapitulated our *in vivo* observations. As expected, PD-1 blockade increased Ly6C expression at both low and high concentrations (p = 0.0071 and 0.0157, respectively) while sPD-L1 supported low Ly6C expression. PD-1 blocking antibodies inhibited the effects of sPD-L1 in a dose-dependent manner (Fig. 5G). Using the same *in vitro* system, we measured oxygen consumption rate (OCR) and found that PD-1 blockade decreased OCR while sPD-L1 increased OCR (Fig. 5H-I). sPD-L1 showed trends towards increased maximum (p = 0.0793) and spare (0.0644) respiratory capacity, while anti-PD-1 blocked the effects of sPD-L1 at low doses and significantly decreased respiratory capacity from baseline at high doses. These data are consistent with our RNA sequencing data showing that sPD-L1 activates PD-1 during monocyte maturation, driving these cells to a restorative phenotype supported by specific metabolic programs.

## 4. Discussion

Revascularization improves outcomes after LVO by limiting primary ischemic injury and is the cornerstone of treatment (Wollenweber et al., 2019; Cagnazzo et al., 2020); however, there are few options to mitigate secondary injury. Post-stroke inflammation plays a central role in secondary injury, but the contributions of immune activation to cerebral edema, neuronal damage, and recovery are incompletely understood (Anrather and Iadecola, 2016; Jayaraj et al., 2019; Shi et al., 2019). Several studies have focused on infiltrating monocytes and monocyte-derived macrophages (Yang et al., 2014; Kanazawa et al., 2017; Tsou et al., 2007). However, these cells are difficult to study *in situ* because they undergo phenotypic changes upon entry into the brain (Garcia-Bonilla et al., 2016; Faustino et al., 2019). Furthermore, subpopulations of monocytes and monocyte-derived macrophages play oppositional roles in recovery. For example, depleting monocytes or blocking their infiltration altogether is counterproductive, as inflammatory or M1-like (Ly6C^hi^) monocyte-derived macrophages transition to a regulatory or M2-like (Ly6C^lo^) phenotype to clear debris and promote healing (Rahman et al., 2017; Kanazawa et al., 2017; Crane et al., 2014). An effective strategy, therefore, could be to reprogram monocytes in the blood before they migrate into the brain. Our data indicate that sPD-L1 acts on monocytes in the periphery to prevent harmful inflammation while promoting tissue repair programs after stroke.

Cerebral edema after stroke is multifactorial. Upregulation of the ion channel SUR1 is a driver of cerebral edema (Simard et al., 2006) and is the target of glyburide, which has been studied in clinical trials for patients with large hemispheric infarctions (King et al., 2018). Immune activation contributes in part by increasing BBB permeability (Huang et al., 2020), although targetable immune pathways have been elusive. In our patient cohort, we found that PD-1 was expressed on blood monocytes and expression levels on intermediate and non-classical monocytes had the strongest correlations with cerebral edema. Although larger patient cohorts are required to determine the potential of PD-1 expression as a biomarker, these data support the potential for clinical translation of our findings in MCAO models and show that PD-1 is differentially expressed on monocyte subtypes after stroke. The latter finding is particularly intriguing as we found the strongest correlations with edema in the intermediate and non-classical subtypes. Intermediate monocytes have been associated with poor clinical outcomes in stroke patients and non-classical monocytes participate in tissue restoration (Kaito et al., 2013). While there are no intermediate monocytes in rodents, we found that sPD-L1 shifted monocytes from an inflammatory to a non-classical subtype. Our human data are consistent with the notion that PD-1 activation on intermediate monocytes may result in an analogous phenotypic switch.

In mice, sPD-L1 administration decreased brain water content by an average of 2.28 % (0.251 mg) at 48 h post-MCAO. These represent biologically significant changes, as previous studies have shown that modest changes in percent water content reflect substantial changes in rodent brain edema (Keep et al., 2012). MRIs obtained at 72 h post-MCAO corroborated these data, as we found that the volume of overall edema (T2 bright) normalized to the volume of infarct (ADC dark/DWI bright) was decreased with sPD-L1 administration. We also measured “excess edema,” which was defined as the volume of hypointensity on ADC maps subtracted from the volume of T2 hyperintensity, to quantify the amount of tissue swelling that occurred outside of the core infarct. Given that differences in survival were driven primarily by deaths within the first 48 h post-MCAO, it is likely sPD-L1 administration decreased intracranial pressure. If this is the case, patients with large infarcts who are at risk of malignant cerebral edema are likely to derive the greatest benefit from peripheral PD-1 activation.

The current treatment for refractory malignant cerebral edema is decompressive hemicraniectomy, which is effective in preventing mortality after large territory infarction but fails to benefit functional recovery (Lin and Frontera, 2021; Vahedi et al., 2007). Conversely, mechanical thrombectomy improves functional outcomes, but can exacerbate inflammation in the setting of reperfusion injury (Ng et al., 2021; Mizuma et al., 2018). These clinical observations suggest that functional outcomes are primarily driven by the extent of the ischemic injury, while inflammation plays a larger role in acute edema. Thus, as PD-L1 treatment did not reduce stroke volume, we were not surprised that long-term functional measures were not significantly changed with sPD-L1 treatment. Subgroup analyses, however, suggested a role for PD-1-mediated inflammation in functional recovery. Future studies will determine if there are functional benefits of sPD-L1 administration in strokes involving smaller or primarily subcortical vascular territories.

The 28-point neuroscore is a well-established test to identify sensorimotor deficits (Encarnacion et al., 2011). While typically analyzed as a total composite score, we found that these results were skewed by survival of more debilitated animals in the treatment group surviving the first 48–72 h after MCAO. Therefore, we performed a subgroup analysis based on performance. sPD-L1 had minimal effect on mice below the 50th percentile. At week 1, however, there was a significant proportion of treated mice in the highest quartile that would have presumably been in the second quartile without treatment. These data suggested that deficits due to severe ischemic injury cannot be overcome by limiting inflammation. Animals with more limited ischemic injuries, however, saw a functional benefit from sPD-L1 administration. After week 1, there were no differences in 28-point scores in either group, consistent with previous reports of early 28-point score recovery in rodent MCAO models (Encarnacion et al., 2011). If these findings translate to stroke patients, PD-1 agonists could expedite early mobilization and facilitate participation in physical rehabilitation.

Presuming that the neuroscore readouts were not sufficiently sophisticated to identify subtle long-term neurologic deficits, we administered the DigiGait and open field tests. Gait is a complex and interconnected outcome in rodents, as deficits in one limb can affect the other limbs in unpredictable ways (Lakes and Allen, 2016; Chan et al., 2023). We identified several parameters of locomotor activity that were significantly changed from baseline by MCAO and evaluated those parameters at weeks 1 and 3. Although no single variable reached significance between the treated and untreated groups, most variables favored treatment, with 100 % of the variables in favor of treatment at week 3. We found the same trend with the open field test administered 4 weeks after infarction as 80 % of variables affected by stroke were improved with sPD-L1. Taken together, these data support the notion that PD-1-mediated inflammation plays a modest, but measurable role in late outcomes.

There are conflicting data on the role of the PD-1/PD-L1 axis in stroke. Ren et al. proposed that PD-1 signaling is protective based on the finding that PD-1-deficient mice had larger infarct volumes and evidence of increased immune activation (Ren et al., 2011). Although we did not observe differences in infarct volumes, our data are generally consistent with these findings, as both global and myeloid-specific PD-1 knockout mice trended toward more edema than wild-type animals. Our data further demonstrated that PD-1 signaling on monocytes skews towards a non-inflammatory program. Taken together, these data indicate that PD-1 is upregulated upon activation of inflammatory monocytes or their precursors and has an inhibitory effect when it binds its soluble ligands. Conversely, global knockout of PD-L1 and PD-L2 as well as PD-L1 blockade have been reported to decrease infarct volumes, reduce monocyte and CD4 + T cell infiltration, and improve neurologic outcomes (Bodhankar et al., 2015; Bodhankar et al., 2013). We found that PD-1 is upregulated on infiltrating monocytes after ischemic stroke, and that the frequency of PD-1+ monocytes in the brain is decreased with systemic sPD-L1 administration. Of note, PD-1+ macrophage/monocyte frequency was not different in the brain or the blood between the groups. This finding is likely due to transient PD-1 expression during phenotype switching, which is a well-known quality of monocytes (Canè et al., 2019). Thus, our tissue-specific knockout experiments that identify monocytes as the site of action of sPD-L1 afford an important insight into monocyte biology. These data should help focus future studies that delineate the kinetics of PD-1 expression on myeloid precursors, monocytes, and macrophages after stroke.

One explanation for the differential effects of PD-1 and PD-L1 ablation is that the context and location of PD-1/PD-L1 binding is important. Although the mechanisms of signaling downstream of PD-L1 are incompletely understood, evidence from cancer indicates that PD-L1 signals independently of PD-1 to promote cell survival (Patsoukis et al., 2020). It is plausible, therefore, that PD-L1 ablation or blockade reduces survival of inflammatory immune cells. Our data support differential effects of PD-L1 based on context with the preponderance of data in favor of peripheral PD-1 activation. Future experiments are required to determine if PD-L1 serves a similar function on circulating myeloid cells in stroke as in cancer. Similarly, soluble PD-L1 has contextdependent activity that determines its effects in health and disease. Our data suggest that soluble PD-L1 may specifically affect maturation of myeloid cells by driving PD-1 signaling. Membrane-bound PD-L1 canonically interacts with PD-1 on lymphocytes in trans, which occurs concurrently with B7 molecules binding CD28 and peptide-loaded MHC molecules binding T cell receptors. Co-stimulatory signals are required for T cell inhibition as PD-1 activation blocks signaling downstream of the TCR and CD28 via SHP2 phosphatase activity (Patsoukis et al., 2020). In this context, sPD-L1 can block PD-1 ligating membrane-bound PD-L1 to paradoxically activate T cells (Song et al., 2011). Monocytes, however, are not subject to TCR and CD28 regulation and our data show that sPD-L1 acts as a PD-1 agonist in this context to reprogram these cells to a patrolling phenotype *in vivo* and *in vitro*. Although the specific effects of sPD-L1 by cell type are not fully understood, our data support the possibility that sPD-L1 might be a clinically viable PD-1 agonist for treating acute inflammation due to selective activity on PD-1 expressing myeloid cells.

In humans, circulating classical, pro-inflammatory CD14^hi^, CD16^lo^ monocytes (which correspond to Ly6C^hi^, CD43^lo^ in mice) are increased during the acute phase of stroke, while the proportion of non-classical, anti-inflammatory CD14^lo^, CD16^hi^ monocytes (Ly6C^lo^, CD43^hi^ in mice) are decreased (Kaito et al., 2013). Our single-cell transcriptomic analysis of circulating monocytes similarly identified two populations of CD11b + monocytes that differentially expressed Ly6C, CD43, and CCR2. Cell clusters predominantly populated by untreated monocytes displayed stronger expression of Ly6C and CCR2 and weaker expression of CD43 (consistent with a classical phenotype), whereas clusters predominantly populated by monocytes from treated mice displayed stronger expression of CD43 and weaker expression of Ly6C and CCR2 (consistent with a non-classical phenotype). Of note, CCR2 expression levels were slightly higher with treatment across clusters. This heterogeneity is consistent with previous literature on CCR2 expression and outcomes after stroke as retention of CCR2 expression on monocyte precursors is important for post-stroke recovery while CCR2 on mature monocytes does not have the same effect (Pedragosa et al., 2020). PD-L1 treated monocytes also displayed significant negative enrichment for inflammatory and defense pathways, and positive enrichment for metabolic pathways. Furthermore, our data show that PD-1 blockade decreases oxidative phosphorylation in opposition to sPD-L1 *in vitro*. As increased oxidative phosphorylation is a hallmark of M2 macrophage polarization, these data confirm functional effects of sPD-L1 on monocyte maturation to a restorative phenotype (Viola et al., 2019) and further support sPD-L1 acting as a PD-1 agonist in opposition to PD-1 blocking antibodies.

It is important to note that the effects of PD-1 signaling on monocytes appears to be disease-dependent as there is evidence that PD-1 blockade augments the influx of monocytes into the brain in Alzheimer’s disease (AD) models (Rosenzweig et al., 2019; Baruch et al., 2016; Ghareghani and Rivest, 2023). In this context, intraperitoneal injection of anti-PD-L1 antibody increased trafficking of Ly6C^hi^ CD45^hi^CD11b+ cells from the periphery into the brain (Rosenzweig et al., 2019; Baruch et al., 2016) mediated by CCR2 signaling (Ben-Yehuda et al., 2021). In contrast, we found that PD-1 activation with sPD-L1 had modest impact on monocyte recruitment, but instead initiated a subset switch in the periphery after stroke. Taken together these findings highlight the importance of context when considering targets in acute inflammation versus chronic or degenerative disease states. With the former, the therapeutic benefit may lie in “defusing” the early and massive influx of proinflammatory monocytes; with the latter, the incoming Ly6C+ monocyte-derived macrophages likely arrive over a longer period of time, and comprise various activation states with disease-modification functions (Rosenzweig et al., 2019), thus there may be a greater benefit to facilitating their migration into the CNS. Future studies are required to determine when and how PD-1 modulation should be optimally applied in acute and chronic diseases.

The Ly6C^hi^, CD43^lo^, CCR2+ phenotype has been previously described as a proinflammatory subset of monocytes that is recruited early to the ischemic brain tissue, while the nonclassical Ly6C^lo^, CD43^hi^, CX3CR1+ phenotype is less responsive to inflammatory stimuli (Miró-Mur et al., 2016). These blood-derived monocytes and macrophages (Mo/Mf) have been shown to accumulate sequentially in the ischemic brain (Garcia-Bonilla et al., 2016), with an influx of Ly6C^hi^ Mo/Mf into the infarct core 3–5 days post-MCAO, and perivascular Ly6C^lo^ Mo/Mf in the surrounding penumbra. Importantly, the accumulation of Ly6C^lo^ monocytes in the surrounding brain is due to a phenotypic switch (or further maturation) of Ly6C^hi^ monocytes, rather than recruitment of a separate monocyte population (Garcia-Bonilla et al., 2016; Miró-Mur et al., 2016).

Our data indicate that PD-1/PD-L1 signaling plays a role in this maturation process. Strauss et al. previously reported that myeloid-specific PD-1 ablation caused increased accumulation of Ly6C^hi^ monocytes in subcutaneous flank tumors (Strauss et al., 2020). More recently, the same group demonstrated that myeloid-specific knockout of the tyrosine phosphatase SHP-2 inhibited maturation and resulted in increased infiltration of Ly6C^hi^ monocytes into subcutaneous tumors (Christofides et al., 2023). Thus, both PD-1 and SHP-2 signaling appear to have critical roles in myeloid lineage fate commitment (Garcia-Bonilla et al., 2016; Miró-Mur et al., 2016). When we compared the differentially expressed genes from our wild-type mice against those published from *SHP-2* knockout mice, we found negative enrichment for over-expressed genes after *SHP-2* knockout (p = 1.149e-24), and positive enrichment for under expressed genes (p= 3.725e-45). These results indicate activation of canonical downstream pathways with PD-L1 administration. A separate differential expression analysis of treated versus non-treated monocytes using M1 and M2 macrophage markers curated from the literature (Szulzewsky et al., 2015) showed significant negative enrichment of M1 markers (p = 0.0016) (Suppl. Fig. 5). Though it should be noted that not all were monocyte-specific markers, the skew away from M1-associated markers may indicate an M2-type lineage commitment for PD-1 activated monocytes. Taken together, these data suggest that PD-1 binding to PD-L1 switches monocyte fates away from pro-inflammatory or classical phenotypes, and toward non-classical, anti-inflammatory phenotypes prior to tissue infiltration. Further studies will be needed to determine whether the Ly6C^lo^ monocytes in the circulation after PD-L1 treatment eventually traffic to the brain to serve a supportive role. The long-term functional outcomes in our studies suggest this is likely the case, and at minimum demonstrate that PD-1/PD-L1 binding on blood monocytes can improve early outcomes without compromising long-term recovery, making peripheral PD-1 activation an intriguing approach to limit inflammatory injury after large territory stroke.

## Supplementary Material

Supplementary data 1

Supplementary image 4

Supplementary image 2

Supplementary image 1

Supplementary image 5

Supplementary image 3

## Figures and Tables

**Table 1:** Patient demographics and clinical characteristics.

Characteristic	No. (%) or Mean ± SD, median (IQR)
Sex, female	9 (64)
Age, yrs	61.6 ± 17.4, 65.5 (24.5)
Large vessel occlusion	
Left MCA	9 (64)
Right MCA	5 (36)
ASPECTS on arrival (n = 13)*	
5	1 (7)
6	1 (7)
7	1 (7)
8	3 (21)
9	4 (29)
10	3 (21)
NIHSS score on arrival	16.9 ± 5.0, 18.0 (8.5)
tPA administered	9 (64)
Time from last known well to presentation, hrs	6.8 ± 5.6, 4.7 (6.5)
Mechanical thrombectomy performed	12 (86)
Time from last known well to recanalization, hrs	8.8 ± 6.3, 7.5 (8.1)
TICI score	
2a	1 (8)
2b	5 (42)
2c	2 (17)
3	4 (33)
Stent placed	1 (8)
Edema volume on MRI, cm^3^	84.4 ± 62.3, 72.9 (103.3)
Infarct volume on MRI, cm^3^	61.5 ± 48.4, 58.1 (100.8)
Edema-to-infarct ratio	1.6 ± 0.6, 1.4 (0.7)

ASPECTS: Alberta stroke program early CT score; ICA: internal carotid artery; NIHSS: National Institutes of Health Stroke Scale; TICI: thrombolysis in cerebral infarction; tPA: tissue plasminogen activator.

* Number in parentheses represents patients without missing data.

**Table 2:** Digigait test results at week 1 after MCAO. Listed by paw are the variables that showed both significance between the three groups on the overall Kruskal Wallis test and a significant difference between the sham group and the untreated stroke group. Therefore, all the listed variables are the gait parameters affected by stroke. Significance was determined by a p value < 0.05 after a Bonferroni correction for multiple comparisons.

	Overall Significance	Sham (median, IQR)	Untreated Stroke (median, IQR)	Sham vs Untreated Stroke	Treated Stroke (median, IQR)	Sham vs Treated Stroke	Untreated vs Treated Stroke
**LEFT FORE PAW**							
Axis Distance	p = 0.018*	−0.75 (−0.83, −0.66)	−0.88 (−0.91, −0.74)	p = 0.021*	−0.84 (−0.93, −0.72)	p = 0.063	p = 0.999
**RIGHT FORE PAW**							
Paw Angle	p = 0.011*	−0.1 (−4.3, 4.2)	6.3 (1.9, 11.4)	p = 0.010*	6.3 (0.5, 9.2)	p = 0.055	p = 0.999
Overlap Distance	p = 0.026*	2.4 (2.2, 3.0)	1.8 (1.1, 2.5)	p = 0.029*	2.1 (1.6, 2.5)	p = 0.085	p = 0.999
**LEFT HIND PAW**							
–	–	–	–	–	–	–	–
**RIGHT HIND PAW**							
%BrakeStride	p = 0.041*	11.1 (10.3, 13.8)	19.1 (12.2, 24.0)	p = 0.040*	12.5 (9.7, 20.4)	p = 0.083	p = 0.830
%BrakeStance	p = 0.041*	18.1 (14.8, 23.5)	28.8 (19.8, 32.2)	p = 0.034*	21.6 (15.7, 32.1)	p = 0.375	p = 0.815
%PropelStance	p = 0.041*	82 (77–85)	71 (68, 80)	p = 0.034*	78 (68, 84)	p = 0.375	p = 0.815
Overlap Distance	p = 0.026*	2.39 (2.15, 2.96)	1.82 (1.09, 2.53)	p = 0.029*	2.10 (1.60, 2.49)	p = 0.085	p = 0.999
Paw Area at Peak Stance	p = 0.004*	0.53 (0.48, 0.56)	0.45 (0.40, 0.51)	p = 0.040*	0.44 (0.36, 0.48)	p = 0.004*	p = 0.999
Paw Area Variability at Peak Stance	p = 0.014*	0.09 (0.07, 0.11)	0.05 (0.03, 0.08)	p = 0.038*	0.05 (0.04, 0.08)	p = 0.021*	p = 0.999
Midline Distance	p = 0.002*	2.34 (2.11, 2.47)	1.74 (1.56, 2.06)	p = 0.002*	1.91 (1.72, 2.15)	p = 0.020*	p = 0.999

**Table 3:** DigiGait test results at week 3 after MCAO. Listed by paw are the variables that showed BOTH significance between the three groups on the overall Kruskal Wallis test AND a significant difference between the sham group and the untreated stroke group. Therefore, all the listed variables are the gait parameters affected by stroke. Significance was determined by a p value < 0.05 after a Bonferroni correction for multiple comparisons.

	Overall Sig	Sham (median, IQR)	Untreated Stroke (median, IQR)	Sham vs Untreated Stroke	Treated Stroke (median, IQR)	Sham vs Treated Stroke	Untreated vs Treated Stroke
**LEFT FORE PAW**							
Paw Area at Peak Stance	p = 0.029*	0.25 (0.24, 0.28)	0.21 (0.17, 0.24)	p = 0.025*	0.22 (0.17, 0.27)	p = 0.215	p = 0.999
Max dA/dT	p = 0.008*	19.9 (17.8, 24.8)	15.5 (11.9, 18.2)	p = 0.006*	16.5 (13.2, 22.2)	p = 0.143	p = 0.632
**RIGHT FORE PAW**							
Paw Area at Peak Stance	p = 0.034*	0.26 (0.23, 0.28)	0.21 (0.18, 0.25)	p = 0.029*	0.23 (0.20, 0.26)	p = 0.263	p = 0.978
Max dA/dT	p = 0.013*	19.9 (17.2, 26.3)	16.2 (13.3, 18.2	p = 0.014*	18.8 (16.3, 22.5)	p = 1.000	p = 0.999
**LEFT HIND PAW**							
Paw Area at Peak Stance	p = 0.002*	0.58 (0.52, 0.61)	0.45 (0.36, 0.52)	p = 0.001*	0.54 (0.41, 0.58)	p = 0.216	p = 0.149
Max dA/dT	p = 0.001*	59.0 (54.4, 67.7)	45.7 (41.1, 54.5)	p = 0.002*	55.6 (49.8, 68.1)	p = 1.000	p = 0.999
Paw Drag	p = 0.001*	−5.47 (−6.72, −4.02)	−2.62 (−3.85, −1.81)	p = 0.001*	−2.71 (−4.82, −2.00)	p = 0.008*	p = 0.999
**RIGHT HIND PAW**							
Paw Area at Peak Stance	p = 0.001*	0.56 (0.52, 0.61)	0.43 (0.36, 0.52)	p = 0.001*	0.50 (0.45, 0.56)	p = 0.136	p = 0.171
Max dA/dT	p = 0.006*	60.7 (49.5, 69.5)	43.1 (37.3, 53.2)	p = 0.005*	51.6 (46.0, 63.3)	p = 0.428	p = 0.192

**Table 4:** Open field test results at week 4 after MCAO. Listed are the variables that showed BOTH significance between the three groups on the overall Kruskal Wallis test AND a significant difference between the sham group and the untreated stroke group. Therefore, all the listed variables are the open field parameters affected by stroke. Reversal of stroke effect with treatment meant that the variable was significantly different between sham mice and untreated stroke mice, but for the treated stroke mice the median returned closer to the sham group median and was no longer significantly different. Significance was determined by a p value < 0.05 after a Bonferroni correction for multiple comparisons.

	Overall Sig	Sham (median, IQR)	Untreated Stroke (median, IQR)	Sham vs Untreated Stroke	Treated Stroke (median, IQR)	Sham vs Treated Stroke	Untreated vs Treated Stroke
Center: entries	p = 0.024*	24.5 (20.0, 33.0)	18.0 (11.0, 24.5)	p = 0.020*	22.0 (11.0, 30.0)	p = 0.157	p = 0.999
Center: distance	p = 0.008*	2.2 (1.7, 2.9)	1.4 (1.0, 1.9)	p = 0.006*	1.8 (1.0, 2.6)	p = 0.138	p = 0.559
Neutral: distance	p = 0.001*	10.8 (9.0, 13.4)	6.9 (5.4, 9.0)	p = 0.001*	8.3 (5.7, 12.2)	p = 0.063	p = 0.332
Wall: entries	p = 0.044*	30.5 (20.0, 38.0)	19.5 (12.0, 28.5)	p = 0.038*	23.0 (14.0, 32.0)	p = 0.271	p = 0.999
Max speed	p = 0.005*	0.37 (0.35, 0.41)	0.31 (0.29, 0.37)	p = 0.012*	0.32 (0.29, 0.38)	p = 0.009*	p = 0.999
Center: max speed	p = 0.006*	0.38 (0.37, 0.41)	0.33 (0.29, 0.38)	p = 0.023*	0.33 (0.28, 0.36)	p = 0.007*	p = 0.999
Neutral: entries	p < 0.001*	125.5 (111.0, 146.0)	91.5 (74.5, 110.5)	p = 0.001*	95.0 (72.0, 124.0)	p = 0.009*	p = 0.999
Neutral: max speed	p < 0.001*	0.42 (0.40, 0.46)	0.34 (0.33, 0.38)	p < 0.001*	0.36 (0.34, 0.41)	p = 0.002*	p = 0.999
Neutral: time mobile	p = 0.005*	140.9 (127.4, 159.1)	98.5 (63.0, 140.6)	p = 0.015*	83.8 (68.6, 137.2)	p = 0.008*	p = 0.999
Wall: max speed	p < 0.001*	0.43 (0.41, 0.47)	0.37 (0.33, 0.40)	p = 0.003*	0.36 (0.33, 0.41)	p = 0.001*	p = 0.999

## Data Availability

Data will be made available on request.
