# Supplementary material for: Soluble PD-L1 reprograms blood monocytes to prevent cerebral edema and facilitate recovery after ischemic stroke
Source: Brain Behav Immun. Author manuscript; Available in PMC 2024 Jul 3. (PMC11220828; doi:10.1016/j.bbi.2023.12.007)
Supplement: Supplementary data 1 [file NIHMS2002822-supplement-Supplementary_data_1.docx]

**Suppl. Fig. 1.** Human PD-1+ blood monocytes: Representative flow cytometry plots to illustrate gating strategy for monocytes (CD11b+CD15-CD19-CD3-)

**Suppl. Fig. 2.** Murine PD-1+ infiltrating brain macrophages: Representative flow cytometry plots to illustrate gating strategy for PD-1+ macrophages (PD1+CD11b+CD45hi).

**Suppl. Fig. 3:** 28 neuroscore test results for wild type mice: Violin plots comparing the raw neuroscores of sham, untreated, and treated mice at weeks 1, 2, 3, and 4. **** indicated p<0.0004

**Suppl. Fig. 4.** Behavioral and immunologic effects of PD-L1 treatment in sham operated mice. (A) PD-L1 treated (n = 15) and untreated (n = 14) mice showed no difference in 28 point scores 24 hours or 7 days after surgery (p =0.837 reflects the association between PD-L1 and have a 28-point neuroscore in the 4^th^ quartile). (B) PD-L1 treatment did not change the density or PD-1 expression of monoyctes in the brain or blood of sham animals. Statistical analyses were performed by Students T-test and Chi-square and Fisher exact tests.

**Suppl. Fig. 5.** PD-L1 expression in treated and untreated mice after MCAO. (A) UMAP of PD-L1 expression (B) Volcano plot showing upregulation of PD-L1 (p-value = 3.06369328080052e-290 with a log2 fold change of 2.14749020509468) and PD-L2 (8.8806260334966e-155 with a log2 fold change of 2.92326622611452) increased in PD-L1 treated cells in cluster 5.

**Data File 1.** Differentially expressed genes for treatment versus no treatment

**Data File 2.** Differential gene expression for monocyte single cell clusters

**Table S1.** 28-point score quartile breakdown in MCAO versus MCAO + PD-L1 wild-type mice, with P-value comparisons for falling in the highest quartile.

|  | Week 2 Quartiles | | | |  |
| --- | --- | --- | --- | --- | --- |
|  | 1 | 2 | 3 | 4 | P-value |
| MCAO | 7 (21) | 6 (18) | 7 (21) | 13 (39) | 0.284 |
| MCAO + PD-L1 | 7 (23) | 7 (23) | 9 (29) | 8 (26) |  |
|  | Week 3 | | | |  |
| MCAO | 8 (24) | 4 (12) | 11 (33) | 10 (30) | 0.524 |
| MCAO + PD-L1 | 7 (23) | 4 (13) | 13 (42) | 7 (23) |  |
|  | Week 4 | | | |  |
| MCAO | 6 (19) | 3 (9) | 17 (53) | 6 (19) | 0.187 |
| MCAO + PD-L1 | 6 (19) | 7 (23) | 8 (26) | 10 (32) |  |

**Table S2.** 28-point score quartile breakdown in MCAO vs. MCAO + PD-L1 PD-1 knockout mice, with P-value comparisons for falling in the highest quartile.

|  | Week 2 Quartiles | | | |  |
| --- | --- | --- | --- | --- | --- |
|  | 1 | 2 | 3 | 4 | P-value |
| MCAO | 3 (13) | 6 (26) | 4 (17) | 10 (43) | 0.618 |
| MCAO + PD-L1 | 7 (26) | 6 (22) | 5 (19) | 9 (33) |  |
|  | Week 3 | | | |  |
| MCAO | 5 (22) | 5 (22) | 6 (26) | 7 (30) | 0.488 |
| MCAO + PD-L1 | 5 (19) | 8 (31) | 3 (12) | 10 (38) |  |
|  | Week 4 | | | |  |
| MCAO | 4 (17) | 5 (22) | 5 (22) | 9 (39) | 0.311 |
| MCAO + PD-L1 | 5 (21) | 7 (29) | 6 (25) | 6 (25) |  |

**Table S3.** 28-point score quartile breakdown in MCAO vs. MCAO + PD-L1 PD-1 myeloid-specific knockout mice, with P-value comparisons for falling in the highest quartile.

|  | Week 2 Quartiles | | | |  |
| --- | --- | --- | --- | --- | --- |
|  | 1 | 2 | 3 | 4 | P-value |
| MCAO | 1 (20) | 2 (40) | 2 (40) | 0 (0) | 0.592 |
| MCAO + PD-L1 | 1 (14) | 3 (43) | 1 (14) | 2 (29) |  |
|  | Week 3 | | | |  |
| MCAO | 0 (0) | 1 (50) | 1 (50) | 0 (0) | 0.999 |
| MCAO + PD-L1 | 1 (25) | 1 (25) | 1 (25) | 1 (25) |  |
|  | Week 4 | | | |  |
| MCAO | 0 (0) | 0 (0) | 2 (100) | 0 (0) | 0.999 |
| MCAO + PD-L1 | 1 (25) | 1 (25) | 1 (25) | 1 (25) |  |

**Table S4.** Antibodies used for flow cytometry of human blood samples

| Antibody (Flow) | Clone | Fluorophore | Dilution | Company |
| --- | --- | --- | --- | --- |
| CD45 | HI30 | AF700 | 1:100 | Biolegend |
| CD11b | ICRF44 | BV421 | 1:100 | Biolegend |
| CD14 | HCD14 | APC-Cy7 | 1:50 | Biolegend |
| CD16 | 3G8 | PE-Cy7 | 1:50 | Biolegend |
| PD-1 | EH12.2H7 | PE | 1:100 | Biolegend |
| CD3 | SK7 | PercpCy5.5 | 1:20 | Biolegend |
| CD15 | H198 | FITC | 1:50 | Invitrogen |
| CD19 | HIB19 | FITC | 1:100 | Biolegend |
| CD11c | 3.9 | BV650 | 1:100 | Biolegend |
| CD8 | Sk1 | BV605 | 1:100 | BD Biosciences |
| Tim-3 | F38-2E2 | APC | 1:100 | Biolegend |

**Table S5.** Antibodies used for flow cytometry of murine tissue samples

| Antibody (Flow) | Clone | Fluorophore | Dilution | Company |
| --- | --- | --- | --- | --- |
| CD3 | 145-2C11 | PercpCy5.5 | 5:200 | Biolegend |
| CD45 | 30-F11 | APC-Cy7 | 1:200 | Biolegend |
| CD11b | M1/70 | AF700 | 1:200 | Biolegend |
| CD11c | N418 | APC | 1:200 | Biolegend |
| Ly6C | HK1.4 | FITC | 1:200 | Biolegend |
| Ly6G | 1A8 | BV650 | 1:200 | Biolegend |
| CD43 | S11 | PE-Cy7 | 1:200 | Biolegend |
| PD-1 | RMP1-30 | BV421 | 1:200 | Biolegend |
| CCR2 | SA203G11 | PE | 1:200 | Biolegend |

**Supplementary Methods**

MRI acquisition parameters. The parameters for T2w images were 36 interleaved coronal 0.5 mm slices that were obtained with a FOV (field-of-view) of 1.5x1.5 cm, image size 128x128, TR of 4,000 ms, TE of 50 ms, 4 averages, and band width 50 kHz. Fat saturation and triggering were used to suppress signal from fat and minimize breathing motion artifacts, respectively. Bruker standard RARE sequence was used with a factor = 8.

The parameters for EPI-DWI Images of 36 interleaved coronal 0.5 mm slices were obtained with a FOV (field-of-view) of 1.5x1.5 cm, image size 128x128, TR of 5,000 ms, TE of 27.76 ms, 4 segments, and a band width 40 kHz, and b-value of 1,500 s/mm2 with 16 gradient directions. EPI-DTI. Fat saturation and triggering were used to suppress signal from fat and minimize breathing motion artifacts, respectively.

Gait Analysis

The DigiGait Imaging System (Mouse Specifics, Boston, MA) was used for gait capture and analysis. The mouse-specific camera and treadmill enclosure were used. Before each run, the clarity of the image capture was optimized for paw capture. The paws were not painted or altered. Customized overhead white LED strip lights were placed on the top of the mouse treadmill enclosure to increase the contrast between the background and the paws. A speed of 10cm/s was used at 1-week post-ischemia and 15cm/s at 3-week post-ischemia. These speeds were determined using training cohorts of post-ischemic mice. The highest speed possible was used such that at least 75% of mice at each time point could adequately maintain gait at that speed for at least 10 steps. Before official image capture, mice were allowed at least one training run on the treadmill to acclimate to the speed and task. Mice were allowed up to three attempts over two trials on the day of recording to maintain consistent gait for at least six strides (six steps with each paw), but ideally ten. The quality of the paw capture and step tracings were evaluated in real time and adjustments were made to noise filters and other parameters before being satisfied with the recording. At least three segments of at least 6-10 steps (without stumbling, contacting a wall or bumper, or turning) each were recorded before capture was completed. Mice who could not satisfy these requirements were excluded from that time point.

For the analysis portion, for each mouse the entire captured video was reviewed. The highest quality segment with at least 6-10 steps was selected. Noise filtering was performed in the Digigait software. Once paw tracings were produced, the actual recording was rewatched and correlated to the tracings to ensure noise was not captured as a step (e.g., the tail or genitalia were not counted as paw) and that steps were not missed. This was corrected manually if possible, including truncating that step if needed. Care was taken not to “over-filter” the tracings such that potential differences in gait pattern were not eliminated. If there were errors that could not be corrected, an alternate segment of video capture was trialed. If the same issue arose, that mouse was excluded. As a quality assessment, the subjective quality of the final tracings was reviewed, as well as calculated parameters, such as mouse length. The indices calculated by the software for good quality mice were then exported for analysis. Indices definitions are provided in prior literature(61, 65).

Single Cell RNA Sequencing

Cell counts and viabilities were determined using the Cell Countess 3 with trypan blue staining. A maximum volume of 77.4uL/sample was used for processing to target up to 10,000 cells. Cells were combined with RT reagents and loaded onto 10X Next GEM Chip N along with 5’ HT gel beads. The NextGEM protocol was run on the 10X Chromium X to create GEMs (gel bead in emulsion), composed of a single cell, gel bead with unique barcode and UMI primer, and RT reagents. Approximately 180uL of emulsion was retrieved from the chip, split into 2 wells, and incubated (45 min at 53°C , 5 min at 85°C, cool to 4°C), generating barcoded cDNA from each cell. The GEMs were broken using Recovery Agent and cDNA was cleaned, following manufacturer’s instructions using MyOne SILANE beads. cDNA was amplified for 11-16 cycles (45 sec @ 98°C, X cycle: 20 sec @ 98°C, 30 sec @ 63°C, 1 min @ 72°C; 1 min @ 72°C, cool to 4°C). Samples were cleaned using 0.6X SPRIselect beads. Quality control (QC) assays were completed using Qubit and Bioanalyzer to determine size and concentrations. 20uL of amplified cDNA was carried into library preparation. Fragmentation, end repair, and A-tailing were completed (5 min @ 32°C, 30 min @ 65°C, cool to 4°C), and samples were cleaned up using double sided size selection (0.6X, 0.8X) with SPRIselect beads. Adaptor ligation (15 min @ 20°C, cool to 4°C), 0.8X cleanup, and amplification are performed, with PCR using unique i7 and i5 index sequences. Libraries underwent a final cleanup using double sided size selection (0.6X, 0.8X) with SPRIselect beads. Library QC was performed using Qubit, Bioanalyzer, and KAPA library quantification qPCR kit. Libraries were sequenced on the Illumina NovaSeq 6000 using v1.5 kits, targeting 50K reads/cell, at read lengths of 28 (R1), 10 (i7), 10 (i5), 91 (R2). Demultiplexing and FASTQ generation was completed using Illumina’s BaseSpace software.

The binary base call (bcl) sequence files from the NovaSeq6000 (Illumina) were converted to FASTQ sequence files using BCL Convert Software (Illumina; bcl2fastq2 v2.20). The FASTQs corresponding to each library from the sequencing run were aligned and count matrices were generated using the Cell Ranger count function (10X Genomics; version cellranger-7.0.0). This matrix included single-cell RNA counts from the two sorted compartments of each of the PD-L1 treated and untreated monocytes.
