# Supplementary figures and images for: Soluble PD-L1 reprograms blood monocytes to prevent cerebral edema and facilitate recovery after ischemic stroke
Source: Brain Behav Immun. Author manuscript; Available in PMC 2024 Jul 3. (PMC11220828; doi:10.1016/j.bbi.2023.12.007)

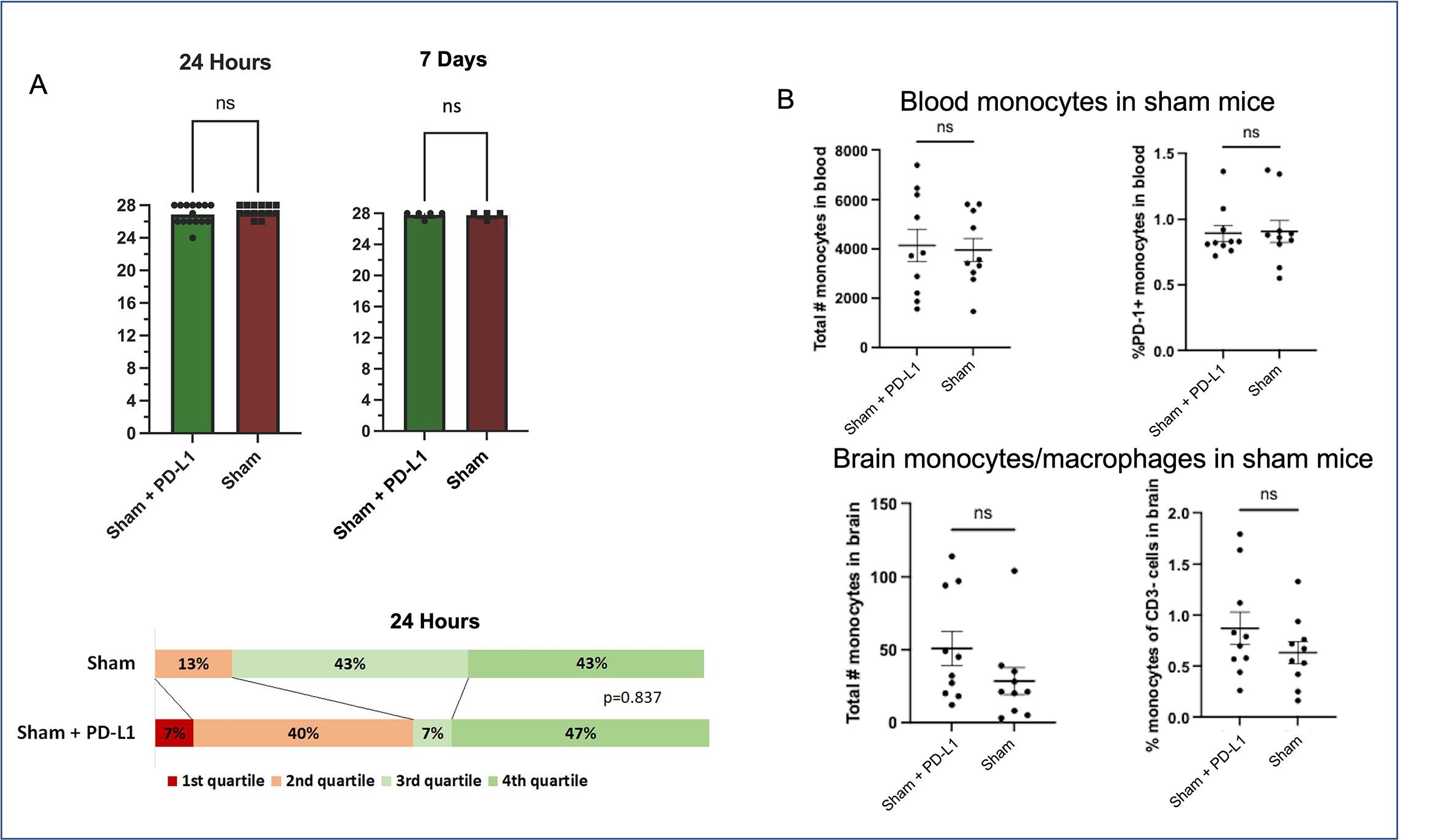

Supplement: Supplementary image 4 [file NIHMS2002822-supplement-Supplementary_image_4.jpg]

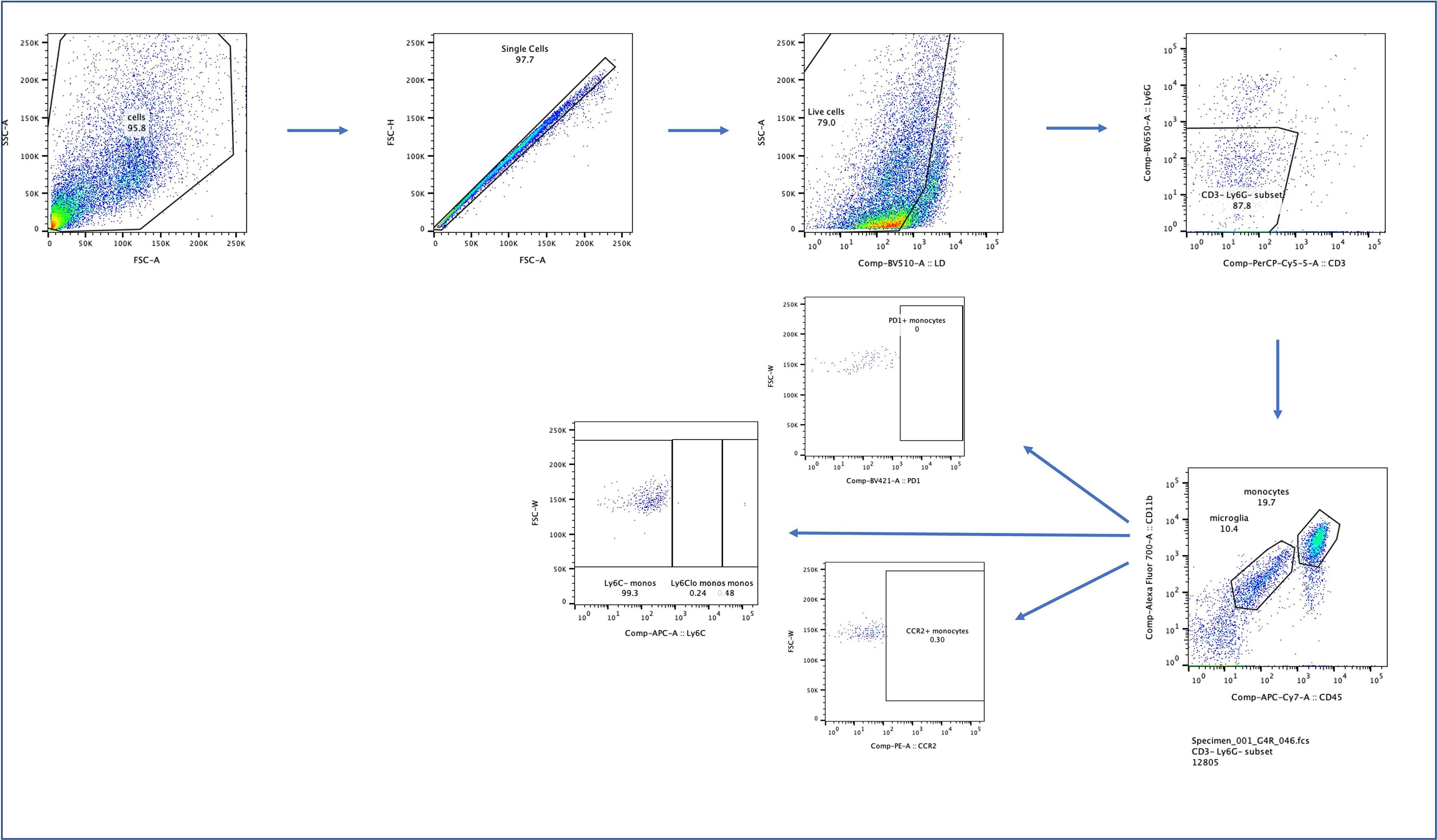

Supplement: Supplementary image 2 [file NIHMS2002822-supplement-Supplementary_image_2.jpg]

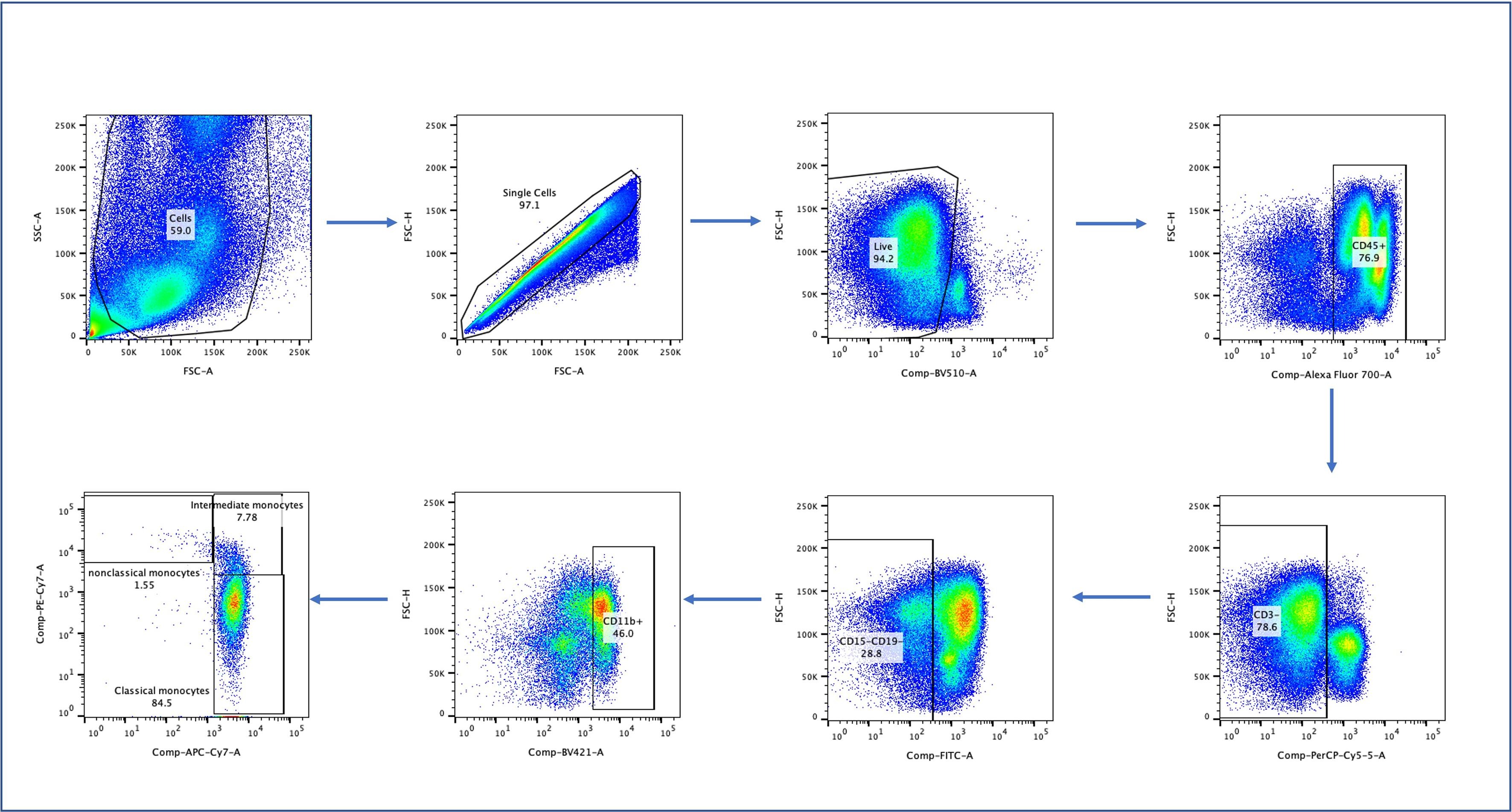

Supplement: Supplementary image 1 [file NIHMS2002822-supplement-Supplementary_image_1.jpg]

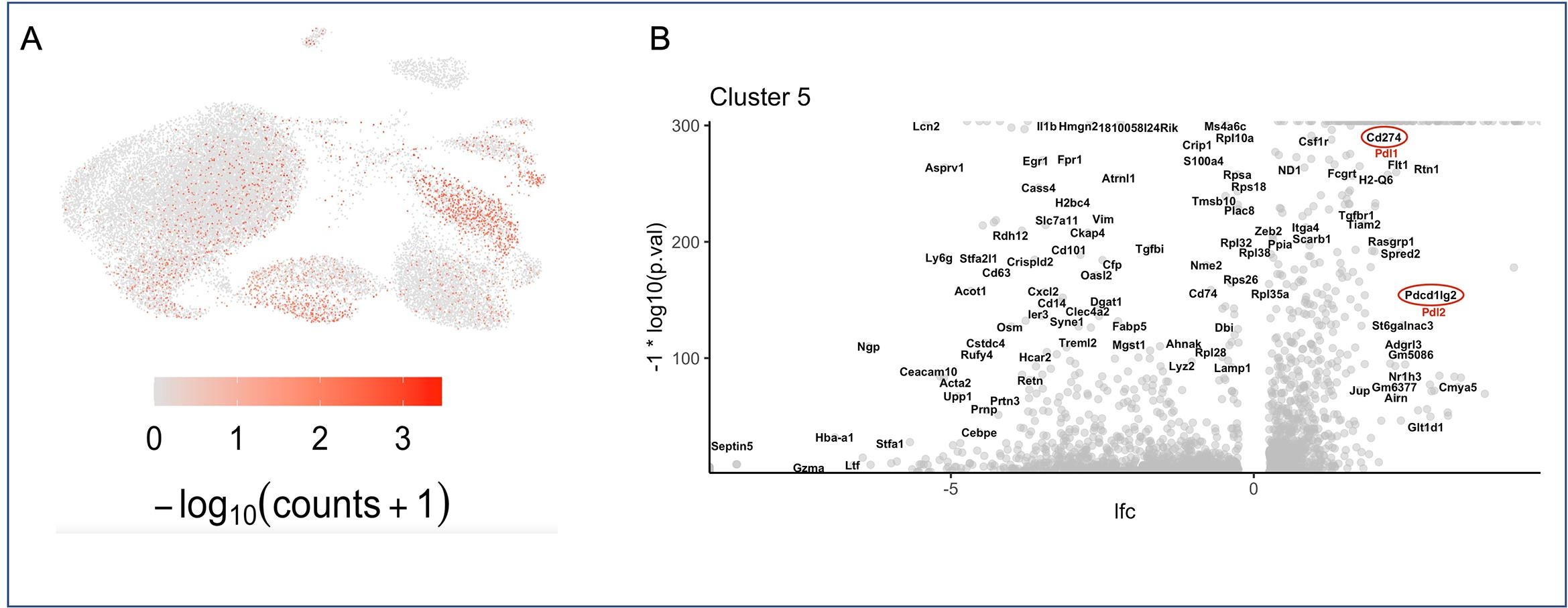

Supplement: Supplementary image 5 [file NIHMS2002822-supplement-Supplementary_image_5.jpg]

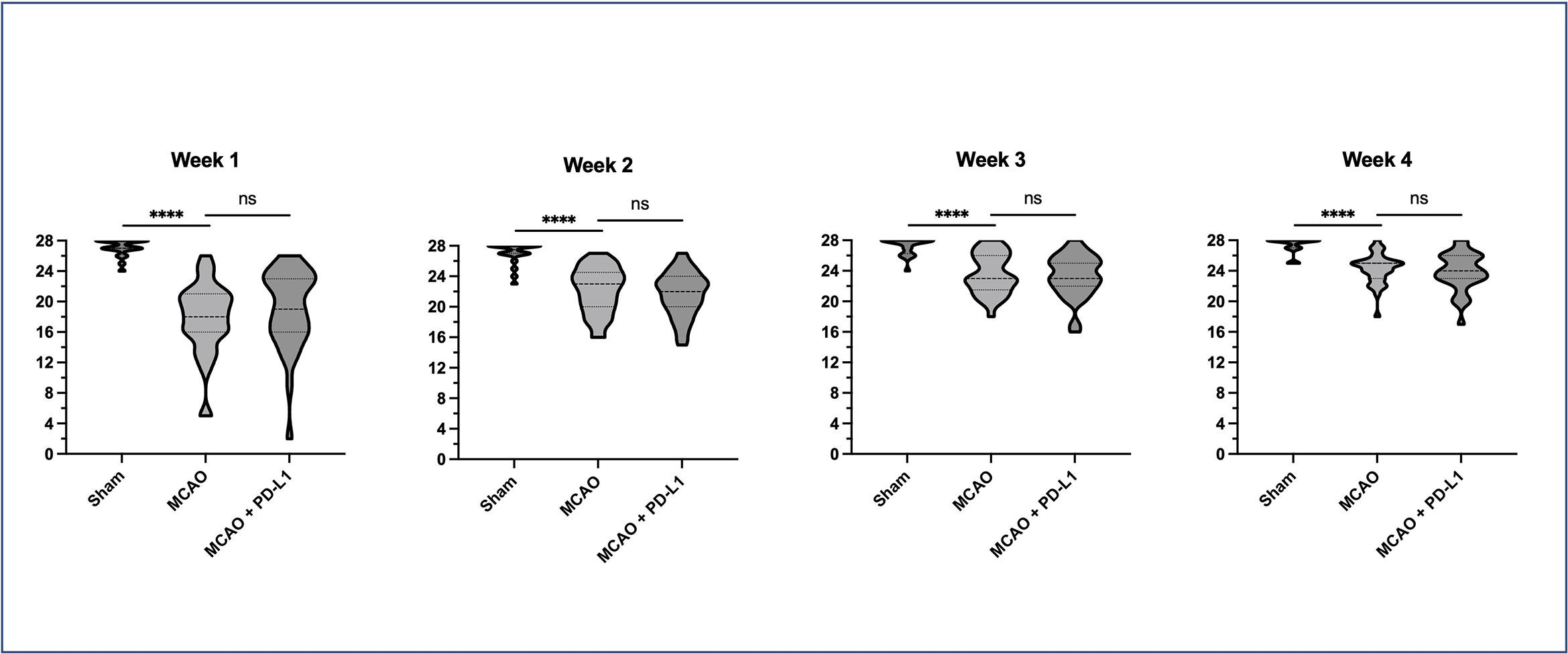

Supplement: Supplementary image 3 [file NIHMS2002822-supplement-Supplementary_image_3.jpg]
